# Maternal Postpartum Work Resumption Stress: Questionnaire Development and Validation

**DOI:** 10.1177/10731911241246607

**Published:** 2024-04-27

**Authors:** Ana Okorn, Madelon L. M. van Hooff, Antonius H. N. Cillessen, Roseriet Beijers

**Affiliations:** 1 Radboud University, Nijmegen, The Netherlands; 2 Open Universiteit, Heerlen, The Netherlands; 3 Radboud University Medical Center, Nijmegen, The Netherlands

**Keywords:** maternal stress, work resumption, postpartum, questionnaire development, validation

## Abstract

Transitioning back to work after maternity leave is increasingly common. While differences exist, for many mothers this transition represents a stressor. This study aimed to define the construct of maternal postpartum work resumption stress and develop and validate a self-report measure in a low-risk sample of Dutch mothers. First, the item pool (*N* = 71) and face and content validity of the questionnaire were established. Next, two independent samples of mothers returning to work (*N* = 298, *N* = 291) were recruited to identify factor structure, reduce the number of items, and assess the dimensionality, reliability, convergent and discriminant validity of the questionnaire. Based on exploratory and confirmatory factor analyses, the reliable and valid REturn to Work INventory (REWINd) with 30 items across three factors was established. While further validation is needed, REWINd can be used to further study the nature and consequences of maternal postpartum work resumption stress.

While welcoming a new baby into their lives is a joyful and gratifying experience, for many mothers, the postpartum period also represents a time of vulnerability and challenges (Groer et al., 2002). Mothers nurture and nurse their young infants while at the same time recovering from giving birth and dealing with fragmented sleep (Horowitz & Damato, 1999; Jevitt et al., 2012; Kaitz, 2007; Razurel et al., 2011). Moreover, as combining motherhood with employment is increasingly common in modern society, another event many mothers face is the transition back to work after maternity leave, the length of which varies considerably across countries (OECD Family Database, 2022). During this transition, mothers establish a new work–life balance, separate from their infant, and readjust to work. While the experience differs greatly among mothers and positive aspects of returning to postpartum employment are evident, the transition represents a challenge for most mothers (e.g., Millward, 2006; Nichols & Roux, 2004; Spiteri & Borg Xuereb, 2012) and might lead to stress (Horowitz & Damato, 1999; Kaitz, 2007; Razurel et al., 2011), as other major transitions do (Mikal et al., 2013). However, there is currently no definition and measure characterizing the stress mothers experience in response to returning to work after maternity leave. The lack of conceptualization and an available measure limits our understanding of the nature, course, and consequences of maternal postpartum work resumption stress for mothers as well as their families. Therefore, in the current study, an operational definition of postpartum work resumption stress is provided, followed by the development and validation of a maternal postpartum work resumption stress questionnaire in a low-risk sample of Dutch mothers.

## Current State of the Literature on Postpartum Work Resumption

To date, most of what is known about mothers’ experiences when returning to work postpartum comes from qualitative and descriptive studies. From those, many describe reintegrating into the workplace after maternity leave as a challenge. To illustrate, in a study of eight British first-time mothers, women experienced return-to-work dilemmas and struggled with re-proving themselves as both a valued employee and a mother (Millward, 2006). Likewise, based on interviews with nine first-time mothers in Norway, Alstveit and colleagues (2011) concluded that the transition back to work can be critical for the well-being of mothers, as they need to adjust their lives to the tension between work and motherhood. Similarly, Spiteri and Borg Xuereb (2012) found that, although mothers would recommend other women to return to work after maternity leave, a group of 10 first-time Maltese mothers acknowledged that going back to work was challenging and not easy.

However, it is important to note that studies also show that not all mothers experience the transition the same way and that positive experiences can be observed as well. For example, in a recent study of 12 Italian nurses returning to work after a leave of more than 12 months, the combination of work and family upon the return resulted in mothers experiencing both difficulties and gains (Costantini et al., 2022). Likewise, in a study with six Australian occupational therapists, these mothers experienced the return-to-work as largely positive, despite intense personal struggles throughout the transition (Parcsi & Curtin, 2013).

Following from this, while experiencing the transition back to work after maternity leave as challenging and stressful seems to be universal across different cultures and policies regarding the length of maternity leave, differences between mothers exist. However, in order to be able to better understand why such differences exists and to more readily identify those mothers who (will) struggle upon their return, a comprehensive assessment of maternal postpartum work resumption stress is needed.

## Conceptualization of Postpartum Work Resumption Stress

The first step in developing a comprehensive assessment is to answer the question what postpartum work resumption stress exactly is and how to define it. In accordance with Lazarus and Folkman (1984), psychological stress is “a relationship between the person and the environment that is appraised by the person as taxing or exceeding his or her resources and endangering his or her well-being” (p. 21). Previous qualitative and descriptive studies show that the transition of returning to work might be appraised by the mother as taxing or endangering, and as such represent a stressor, for several reasons.

First, when returning to work, mothers need to find a new balance between their work and personal life domains. When doing so, mothers might experience thoughts and emotions, such as experiencing role-overload (e.g., Nichols & Roux, 2004; Parcsi & Curtin, 2013), worrying about their social life (e.g., Gallegos, 2007; Morris, 2008), and feeling exhausted (e.g., Spiteri & Borg Xuereb, 2012). Second, when re-joining the workforce, mothers have to separate from their new born for longer periods of time and leave their babies in someone else’s care. This might cause mothers to experience thoughts and emotions, such as guilt for leaving their baby behind (e.g., Millward, 2006; Nichols & Roux, 2004), worrying about missing-out on important milestones in the baby’s development (e.g., Alstveit et al., 2011; Brand & Barreiro-Lucas, 2014), and not feeling in control over their baby’s care and upbringing (Spiteri & Borg Xuereb, 2012). Third, upon their return to work, mothers need to readjust to their work. This might lead mothers to experience thoughts and emotions, such as worrying about their work performance (e.g., Brand & Barreiro-Lucas, 2014; Morris, 2008), worrying that they will be negatively perceived by the people they work with (e.g., Alstveit et al., 2011; Parcsi & Curtin, 2013), and feeling the need to re-prove themselves as a good employee (e.g., Millward, 2006).

As emotions and cognitions instigated by a stressful stimulus (e.g., the transition back to work after maternity leave) are part of a stress response (Crosswell & Lockwood, 2020), experiencing many of above mentioned negative (and few of positive) thoughts and emotions related to returning to work postpartum can be seen as maternal postpartum work resumption stress, which we define here as *an aversive psychological reaction to returning to work postpartum, that disrupts the individual’s homeostasis*. Furthermore, the thoughts and emotions seem to pertain to three domains in which postpartum work resumption stress might manifest itself: (a) the establishment of a new work-life balance, (b) separation from the baby, and (c) work readjustment.

## The Present Study

Next to providing a definition, the goal of this study was to develop and validate a self-report measure of maternal postpartum work resumption stress in a low-risk sample of Dutch mothers. To our knowledge, no such self-report measures exist and this study is the first to develop such a scale. Such a measure will make it possible to further study the course, predictors, and consequences of maternal stress during the transition back to work after maternity leave. The development and validation of our new measure, the REturn to Work INventory (REWINd), followed the three distinct phases recommended by Boateng and colleagues (2018): item development (Phase 1), scale development (Phase 2), and scale evaluation (Phase 3). First, the item pool and face and content validity of the measure were established (Phase 1). Next, two independent samples of mothers returning to work postpartum were recruited to identify the factor structure and reduce the number of items of the questionnaire (Phase 2), and assess the dimensionality and psychometric properties of the questionnaire (Phase 3). For the flow chart depicting the procedure taken to arrive at the final version of the questionnaire, see Figure S1 in Supplementary Material. The study has been reviewed by the Ethics Committee of the Faculty of Social Sciences, Radboud University, and there is no formal objection. Phase 2 and 3 study design, hypotheses, and analyses were pre-registered on AsPredicted before data collection (https://aspredicted.org/wz5e8.pdf, https://aspredicted.org/b7gw8.pdf).

## Phase 1: Item Development

### Item Generation

The first and fourth authors and a student assistant developed 71 new items, from which 62 were inspired by previous qualitative and descriptive studies on postpartum return to work experience (i.e., Alstveit et al., 2011; Beijers et al., 2022; Brand & Barreiro-Lucas, 2014; Gallegos, 2007; Millward, 2006; Morris, 2008; Nichols & Roux, 2004; Parcsi & Curtin, 2013; Spiteri & Borg Xuereb, 2012). Nine items were inspired by existing theoretically relevant measures (i.e., Maternal Separation Anxiety Scale (Hock et al., 1989), Work-Family Conflict Scale (Haslam et al., 2015), and Survey Work-home Interaction-NijmeGen (Geurts et al., 2005)). The items were capturing thoughts and emotions mothers might have when returning to work after maternity leave, from which 28 addressed the re-establishment of a new work–life balance, 23 addressed separation from their baby, and 20 addressed work readjustment.

To ensure that our questionnaire was situation-neutral, and as such appropriate for a broad population, no items were included that referred to thoughts and emotions associated with a specific situation, such as worries about breastfeeding and lack of partner support. To encourage respondents to make a choice and ensure a sufficient opportunity to detect respondents’ true scores, a six-point Likert-type response format, ranging from 1 (*very untrue*) to 6 (*very true*) was used. Moreover, to capture the current state of postpartum work resumption stress, mothers were instructed to rate how true each statement was for them in the past 7 days.

Following the initial item generation, a research assistant, the second and fourth authors, and a practitioner working with mothers combining motherhood with employment evaluated the items based on their relevance (e.g., Does it fit the definition? How would a mother with high/low levels of postpartum work resumption stress rate a certain item), and provided suggestions for additional items and improvements. The questionnaire was adjusted after each evaluation, with 13 items dropped, 5 added, and modifications made to grammar and word choice. This resulted in a set of 63 items.

### Evaluation by Target Population: Face Validity

To ensure that items were perceived as appropriate and meaningful by the target population (i.e., face validity; Holden, 2010), online cognitive interviews were conducted with six Dutch mothers returning to work after maternity leave in the last 2 to 12 months (see Table 1, Phase 1, for demographic characteristics). Mothers were asked to review the questionnaire independently and write down whether any items were unclear, difficult to answer or inappropriate, and whether certain emotions or thoughts were not yet addressed. Their notes were discussed in detail during the online interview, which was led by the research assistant and the first author, audio recorded, and transcribed later.

**Table 1:** Demographic Characteristics of the Samples Used in Phase 1, Phase 2 and Phase 3.

Variable	Phase 1 (*N* = 6)	Phase 2 (*N* = 298)	Phase 3 (*N* = 291)
Age, years	34.8 ± 3.1 (31–39)	31.9 ± 3.8 (21–42)	33.0 ± 3.5 (25–44)
Nationality, % Dutch	100	98.7	98.6 (*n* = 290)
Education, % high	100	83.2	90.0
Relationship status, % with partner	100	98.3	98.3
Number of children			
One child, %	66.7	64.8	62.2
Two children, %	0	27.2	30.9
Three children or more, %	33.3	8.1	6.9
Working hours per week^a^	29.3 ± 4.8 (23–36)	28.4 ± 7.0 (12–91)	29.3 ± 5.7 (15–45)
Between 12 and 24 hours, %		38.9	28.9
Between 24 and 32 hours, %		43.3	54.0
More than 32 hours, %		17.8	17.2
Length of leave, weeks^b^	27.1 ± 19.7 (14–65.3)	19.5 ± 5.7 (10–46)	21.5 ± 7.2 (8–74)
Time back at work, weeks	25.2 ± 18.3 (10–51)	12.2 ± 8.1 (0–30.5)	11.0 ± 7.3 (0.3–30)
Child age at return, weeks	16.0 ± 11.4 (10–39.2)	14.8 ± 4.3 (0–39.1)	14.2 ± 3.9 (3–34)
Child current age, weeks	41.3 ± 18.4 (21.8–60.9)	27.0 ± 8.6 (9–56.6)	26.9 ± 9.1 (8–58.6)
Child sex, % boys	66.7	52.5 (*n* = 295)	54.2 (*n* = 288)
Working from home^c^			
Not working from home, %			29.4 (*n* = 289)
< 50% of the time, %			38.1 (*n* = 289)
> 50% of the time, %			32.5 (*n* = 289)

Note. Nationality (0 = *Dutch*, 1 = *Other*); Education, 0 = *Lower/medium*, 1 = *High* (higher vocational education or scientific/academic education,); Relationship status (0 = *With partner*, 1 = *Without partner*); Number of children (0 = *One child*, 1 = *Two children*, 2 = *Three children* or *more*); Child sex (0 = *Girl*, 1 = *Boy*); Working from home (0 = *0%*, 1 = *0%–25%*, 2 = *25%–50%*, 3 = *50%–75%*, 4 = *75%–100%*).

a The information on women’s employment schedule (e.g., working hours, teleworking) prior to maternity leave were not recorded in our study. ^b^In the Netherlands, mothers are entitled to 16 weeks of leave (4–6 weeks before the due date and at least 10–12 after childbirth; average payment rate of 100%) and fathers are entitled to 6 weeks of leave (average payment rate of 79.9%). The information about the length of leave taken by partners was not recorded within our study. ^c^Amount of time working from home was not measured in Phases 1 and 2.

The transcripts, examined by the first and fourth authors, revealed good face validity of the questionnaire. Mothers found the questionnaire relevant, and the items representative of their experiences when returning to work postpartum. They found most items were easy to understand and appropriate for them. Some suggestions for improvement were given as well, such as a need for a better balance between positively and negatively framed items to provide a more neutral feel of the questionnaire, rewording of some items to provide additional clarity, and deletion and inclusion of some items to better capture their experiences. Based on this feedback, of the 63 items, nine items were dropped, six were added, and modifications to grammar and word choice were made. This resulted in a set of 60 items.

### Evaluation by Independent Review Panel: Content Validity

To ensure that items were relevant and represented the targeted construct well (i.e., content validity; Haynes et al., 1995), a panel of five independent reviewers, four doctoral students and one postdoc in the fields of developmental and occupational health psychology and maternal stress research, rated the relevance of the items (i.e., Does the item represent the domain of interest?) on a four-point Likert-type response format (1 = *not relevant*, 4 = *very relevant*). A modified kappa statistic was calculated to examine the agreement among the reviewers that the item is relevant, with a value above .74 considered evidence of excellent agreement on relevance (Polit et al., 2007). In addition, suggestions for improvement were recorded.

Of the 60 items, 20 had a modified kappa below .74 (range -.03 to .42) and were therefore considered for revision or deletion (Polit et al., 2007). Of these, nine were modified, seven excluded, and four kept because of their strong theoretical relevance. The other 40 items showed excellent agreement on relevance (modified kappa > .74, Polit et al., 2007). Of these, 13 were kept as they were and 26 were slightly modified (i.e., changes in grammar and word choice, or adding a general stem “Now that I am back to work” to the sentence). Moreover, following the final evaluation, one item (*‘Now that I’m back at work, I’m worried that my baby will miss me when he or she is not with me’*) was excluded due to its ambiguity, despite an excellent agreement on relevance. Specifically, longing for someone is believed to be an emotion of a higher-order, one that develops from the primary emotions later in early-childhood (Holm, 1999). As such, maternal disagreement as a response to this item might represent the emotions and thoughts of this mother or her understanding that her infant is not (yet) capable of missing her. This yielded a pool of 52 items (see Table S1 in Supplementary Material), comprising a questionnaire with good face and content validity.

## Phase 2: Scale Development

### Method

#### Participants, Procedures, and Measures

When deciding on the required sample size, we followed the recommendations by Mundfrom and colleagues’ (2005). Their simulation study addressed minimum sample size requirements for conducting factor analyses, and found that in general, when the variables-to-factors ratio exceeded 6, the minimum sample size began to stabilize regardless of the number of factors or the level of communality, and was never greater than 180. Based on this, the sample size of 250 was considered sufficient to achieve adequate power in our analyses, due to the high overdetermination of the expected factors (52 items across three factors), and thus expected variables-to-factor ratio higher than six. Participants were recruited via posts and ads on social media (i.e., LinkedIn, Facebook) and the researchers’ personal network between August and October 2021. The inclusion criteria were: >18 years, working and living in the Netherlands, having one or more children, having returned to work after maternity leave in the last six months, working at least 12 contractual hours per week, being separated from children during the working hours, and having a child who was not a twin, born pre-term, or having developmental or health difficulties.

A sample of 390 participants indicated that they fit the study criteria and completed an online informed consent form. Participants were asked to fill-in an anonymous online questionnaire, including demographic characteristics and the 52-item postpartum work resumption stress questionnaire that resulted from Phase 1. Items were rated on a six-point Likert-type response format, with 18 items reverse coded. From 390 participants, 337 completed the survey. Additional inspection showed that 39 of those did not meet the study criteria (i.e., not yet returned to work or back at work for longer than 7 months, working for less than 12 hours, not providing clear information about when they returned to work). This resulted in a final sample of 298 Dutch mothers returning to work after maternity leave in the last 6 months (see Table 1, Phase 2, for demographic characteristics). As compensation, mothers could leave their email addresses to participate in a raffle for 20 vouchers of €20.

#### Analyses Plan

Preliminary analyses and statistical methods for determining the number of factors were performed in R 4.2.1. As suggested (Carpenter, 2018; Watkins, 2018), to ensure that the data were appropriate for factor analysis, the distributions and the factorability (i.e., sufficient correlations among the items) of the items were examined, by inspecting the item variability, correlation matrix (*lavaan v0.6.12*; Rosseel, 2012), Bartlett’s test of sphericity (*EFAtools v0.4.1*; Steiner & Grieder, 2020), Kaiser–Meyer–Olkin measure of sampling adequacy (*EFAtools v0.4.1*; Steiner & Grieder, 2020) and corrected item-total correlations. Following this stage of data screening, as recommended by Goretzko and colleagues (2021), the number of factors to retain in the factor analysis was determined based on theoretical considerations and two statistical methods specific for this purpose. The first was parallel analysis, based on polychoric correlations, principal components extraction, mean eigenvalue criterion and 1000 replications, as this was found to lead to the most accurate parallel analysis estimations (Garrido et al., 2013). This was carried out using *random.polychor.pa* (*v1.1.4.4*; Presaghi & Desimoni, 2020) and function *polyPA* introduced by Lubbe (2019). The second was the comparison data approach, based on Spearman rank-order correlations, random samples of 10000 cases, alpha level of .30, and 500 replication samples (Ruscio & Roche, 2012). This was carried out using *EFAtools* (*v0.4.1*; Steiner & Grieder, 2020) and *RGenData* (*v1.0*; Ruscio, 2018).

After determining the number of factors to retain, we ran a series of exploratory factor analyses, or more specifically common factor analyses (Carpenter, 2018; Watkins, 2018) in MPlus (version 8.6). Because of the ordinal nature of our data, we used the polychoric correlation matrix and unweighted least squares estimator, which was found to provide more accurate and less variable parameter estimates, and more precise standard errors compared with the diagonally weighted least squares estimator (Forero et al., 2009). Finally, we used oblique rotation (geomin) to allow factors to correlate (Carpenter, 2018).

After establishing a factor structure, items were deleted based on a priori criteria (Carpenter, 2018; Costello & Osborne, 2005). Specifically, with the exception of a theoretical justification to retain, items with factor loadings below .30 (i.e., non-salient loadings), cross-loadings above .30 (i.e., non-trivial cross-loadings), and with communalities below .40 were omitted one-by-one. Moreover, to rule out the weak and unstable factors, only factors with at least three items loading uniquely on the factor were considered (Carpenter, 2018; Costello & Osborne, 2005). To ensure factor reliability, categorical omegas (Green & Yang, 2009) were computed in *R* (*misty v0.4.6*; Yanagida, 2022).

### Results

#### Preliminary Analyses

There were no missing data. All 52 items were rated in the full range from 1 to 6. Although some inter-item polychoric correlations were low, all items correlated moderately with at least a few other items. In addition, with the exception of somewhat high correlations between items 3 and 51 (*r* = .83), 14 and 31 (*r* = .82), 14 and 52 (*r* = .81), and 31 and 52 (*r* = .83), no multicollinearity was observed (see Figure S2 in Supplementary Material). The factorability of the items was further supported by significant Bartlett’s test of sphericity, *χ*^2^(1326) = 9199.51, *p* < .001, and an adequate value (.94) of the Kaiser–Meyer–Olkin measure of sampling adequacy. Moreover, the corrected item-total correlations ranged between .37 and .77, which was in line with the recommendations for a good scale (Ferketich, 1991). Based on this, the data were considered suitable for factor analysis.

#### Number of Factors to Retain

From a theoretical perspective, a three-factor structure was expected for the three aspects of postpartum work resumption stress identified in our substantive literature review: (a) the establishment of a new work-life balance, (b) separation from the baby, and (c) work readjustment. The parallel analysis indicated five factors. The comparison data approach suggested six factors. Based on the guidance and example introduced in Watkins’s systematic, evidence-based guide to conduct EFA (Watkins, 2018), we next evaluated plausible solutions between three and six factors (starting with the highest number) in a series of exploratory factor analyses.

#### Identification of Factor Structure

Geomin rotated loadings, communalities, and categorical omegas of the six-, five-, four-, and three-factor solutions are shown in Tables S1-S4 (Supplementary Material). While solutions with six, five, and four factors were considered inadequate (for detailed explanation see notes under Tables S2-S4), the three-factor solution was found to be adequate. Specifically, at least three items loaded uniquely on each factor; 22 items had the highest loading on the first factor, 16 on the second factor, and 14 on the third factor. Furthermore, all three factors showed good reliability. Given these findings, the three-factor solution was retained for further analyses.

#### Item Deletion

After establishing the factor structure, items were excluded one by one to find a solution in which each item had a salient loading (> .30) on one factor only (i.e., simple structure; Brown, 2015), and was sufficiently related to the other items of the questionnaire (item communality above .40; Costello & Osborne, 2005). First, the items with salient loadings on more than one factor were identified. From those, the item with the lowest highest loading was considered for deletion. In this step, Items 32, 39, 18, 52, 5, 14, 46, 31 and 22 were excluded. While, in accordance with Carpenter’s (2018) guidelines for scale development, an item loading cut-off anywhere between .30 and .40 is supported by their literature review, a cut-off of .32 is recommended. Furthermore, Hair et al. (2009) recommend a cut-off for item loadings relative to sample size, with an item loading of a minimum of .35 and a minimum of .30 needed for significance, for the sample size of 250 and 350, respectively. In line with this, in addition to the pre-registered analyses, exploratory sensitivity analyses with a more strict cut-off criteria (> .325) for the salient item loadings/non-trivial cross-loadings based on our sample size (*N* = 298) were performed. The solution found was the same as the solution found when .30 was used as a cut-off.

Next, items with the communality below .40 were identified and the item with the lowest communality was considered for deletion. In this step, Items 49, 7, 29, 28, 48, 35, 47, 41, 16, 24, and 4 were excluded. Finally, in the last factor solution, some item loadings did not reach the value that is generally considered necessary for practical significance (.50; Hair et al., 2009). Therefore, Items 50 and 27 were additionally excluded, yielding a solution with all factor loadings reaching practical significance.

To ensure that our solution was stable regardless of which oblique rotation is used, the final model was analyzed with oblimin, promax, and quartimin rotations as well (see Tables S5–S7 in Supplementary Material). All analyses yielded the same solution as obtained with geomin rotation, supporting the stability and appropriateness of our final solution.

Table 2 presents the factor loadings and communalities of the final solution. Following the item deletion, 30 items across three factors were retained. Factor 1 was saturated with 12 items with loadings ranging from .59 to .80 (*M* = .69). This factor represented maternal thoughts and emotions related to lack of time, role-overload, and emotional and physical exhaustion when returning to work postpartum, and therefore was labeled *Work-life imbalance*. Factor 2 was saturated with 10 items with loadings ranging from .57 to .91 (*M* = .73). This factor represented maternal thoughts and emotions related to guilt for leaving the baby, worries about the baby’s health, and feelings of not being a good enough parent when returning to work postpartum, and therefore was labeled *Child-related concerns*. Factor 3 was saturated with eight items with loadings ranging from .50 to .99 (*M* = .74). This factor represented maternal lack of positive thoughts and emotions related to returning to work, such as enjoying being back at work and being proud to be a working parent, and therefore was labeled *Lack of enrichment*. The geomin correlations between the factors were positive and moderate: .49, .52, and .54 between factors 1 and 2, 1 and 3, and 2 and 3, respectively. All factors showed good reliability: categorical omega of .93 for factor 1, .92 for factor 2, and .89 for factor 3.

**Table 2:** EFA Results of the Final Solution.

Item	*λ_1_*	*λ_2_*	*λ_3_*	*h^2^*
**Factor 1: Work-Life Imbalance**				
*Now that I am back at work, …*				
45. I feel like I do not have enough time to take care of myself.	.80			.64
8. I feel like I do not get the chance for my hobbies or to do other fun things.	.77			.55
17. I struggle to meet the various responsibilities I have in my life.	.77			.55
37. I struggle managing my time well.	.77			.55
13. it is hard to find time for myself.	.76			.55
34. I feel like everything is chaotic.	.70			.57
6. I feel exhausted.	.69			.54
25. I feel overwhelmed by my role as a working parent.	.63			.59
15. I feel stressed.	.63			.51
2. I worry about myself.	.61			.51
11. I feel like I have the energy to do everything I want.	.60			.52
19. I worry about my social life.	.59			.32
**Factor 2: Child-Related Concerns**				
*Now that I am back at work, …*				
51. I feel guilty for leaving my baby with others.		.91		.81
3. I feel like I am letting my baby down.		.84		.77
9. I am afraid that the bond with my baby will worsen.		.80		.53
20. I am concerned that I will miss out on important moments in my baby’s life.		.77		.56
44. I feel like I am not with my baby often enough.		.74		.60
23. I am afraid that being back at work is not the best for my baby.		.72		.70
30. I am afraid that my baby will get sick, hurt, or upset while I am at work.		.68		.46
40. I feel like I have no control over my baby’s care and upbringing.		.64		.49
33. it feels too early to leave my baby in someone else’s care.		.59		.55
12. I feel like I am falling short as a parent.		.57		.59
**Factor 3: Lack of Enrichment**				
*Now that I am back at work, …*				
38. I enjoy being back at work.			.99	.85
1. I like having contact with people from work again.	-.32		.87	.57
43. I feel proud as a working parent.			.79	.56
10. I feel ready to be back at work.			.73	.76
26. my work gives me the opportunity to recharge myself.			.71	.40
36. I lack the motivation to do my job.			.69	.55
42. my life feels complete.			.61	.44
21. I feel like I have structure in my life.			.50	.40
*ω*	.93	.92	.89	

*Note. λ_1_* = geomin factor loading on the first factor; *λ_2_* = geomin factor loading on the second factor; *λ_3_* = geomin factor loading on the third factor; *h* ^2^ = item communality; *ω* = categorical omega. Non-salient loadings (<. 30) are omitted for clarity.

All items loaded saliently on one factor only, except for Item 1. Item 1 had salient loading on two factors, but the loading on the first factor (-.32) seemed trivial compared with the loading on the third factor (.87) and was thus considered acceptable. All items had a communality of .40 or larger and were thus sufficiently related to one another, except for Item 19. Although the communality of Item 19 was somewhat lower (.32) than recommended (Costello & Osborne, 2005), it was kept in the solution, because of its strong theoretical justification and satisfactory association with the factor to which it belonged (.59).

### Interim Summary and Discussion After Phase 2

A questionnaire with 30 items across three factors, representing maternal postpartum work resumption stress related to *Work–life imbalance*, *Child-related concerns*, and *Lack of enrichment*, was established. The factors capturing postpartum work resumption stress due to the establishment of a new work-life balance (*Work–life imbalance*) and separation from the baby (*Child-related concerns*) were in line with our theoretical expectations. The expected factor measuring maternal postpartum work resumption stress related to work readjustment did not emerge. The reason for this might be that many items proposed to capture postpartum work resumption stress related to maternal work readjustment were not generalizable across all types of jobs and professions. For example, proving oneself at work and focusing and functioning well at work may apply to certain jobs (e.g., with high pressure and responsibility) but not to others. Thus, negative thoughts and emotions about work readjustment may not be a part of postpartum work resumption stress for all mothers.

We did find, however, a factor capturing maternal postpartum work resumption stress related to a lack of positive thoughts and emotions about returning to work (i.e., *Lack of enrichment*). Previous descriptive and qualitative literature indicated that besides experiencing many negative thoughts and emotions about returning to work, most mothers experience positive thoughts and emotions as well. Not experiencing such positive thoughts and emotions when returning to work seems to be an aspect of returning to work postpartum that independently contributes to mothers’ experience of postpartum work resumption stress.

## Phase 3: Scale Evaluation

### Method

#### Participants and Procedures

In accordance with Wolf and colleagues (Wolf et al., 2013), when performing confirmatory factor analysis, the minimal sample size for the three-factor structure, with factor loadings of .50, eight indicators per factor, and moderate factor intercorrelations (.50) is 120. Based on this, the sample size of 250 was considered sufficient to achieve adequate power in our analyses. Participants were recruited through posts and ads on social media (i.e., LinkedIn, Facebook) and the researchers’ personal network between December 2021 and January 2022. The inclusion criteria were the same as for Phase 2. In addition, to prevent an overlap between the second and third sample, mothers were asked whether they had previously participated in our study. Only mothers indicating that they have not taken part in Phase 2 (by clicking that answer) were invited to complete the survey for Phase 3.

A sample of 393 participants indicated that they fit the study criteria and completed an online informed consent form. Of those, 384 indicated that they did not take part in Phase 2 of our study and were thus asked to fill-in an anonymous online questionnaire. While 307 mothers completed the survey, additional inspection showed that 16 of those did not meet the study criteria (i.e., not yet returned to work or back at work for longer than 7 months, working for less than 12 hours, not providing clear information about when they returned to work). This resulted in a final sample of 291 Dutch mothers returning to work in the last six months (see Table 1, Phase 3, for demographic characteristics). As compensation, mothers were able to leave their email addresses to receive a voucher worth €5.

#### Measures

##### Maternal Postpartum Work Resumption Stress

This was measured with the 30-item REturn to Work INventory (REWINd), that resulted from Phase 2. The scale has three subscales representing maternal postpartum work resumption stress related to *Work-life imbalance* (12 items), *Child-related concerns* (10 items), and *Lack of enrichment* (8 items) (see Table 2). Mothers rated how true each statement was for them in the past seven days on a six-point Likert-type response format. Eight items were reverse-coded. Total and subdomain scores were calculated as the mean across items, with higher scores indicating higher levels of stress.

##### Anxiety

Maternal anxiety was measured with the 20-item state anxiety subscale of the State-Trait Anxiety Inventory (STAI; Spielberger, 1983). Mothers rated how they felt at the moment of data collection on a four-point Likert-type response format. The mean across all 20 items was calculated, with higher scores indicating higher levels of anxiety. The reliability of the scale was high, with a categorical omega of 1.00.

##### Postpartum Depression

The Edinburg Postnatal Depression Scale (EPDS; Cox et al., 1987) was used to assess maternal postpartum depression, with 10 items scored on a four-point response format. Mothers were asked to choose an answer that came closest to how they had felt in the past seven days. The mean across all 10 items was computed, with higher scores indicating higher levels of depression. The reliability of the scale was high, with a categorical omega of .89.

##### Perceived Stress

Maternal perceived stress was measured with the Perceived Stress Scale (PPS; Cohen et al., 1983). Mothers rated 14 items on a four-point Likert-type response format, indicating how often they experienced certain feelings or thoughts in the past month. The mean across all 14 items was calculated, with higher scores indicating higher levels of stress. The reliability of the scale was high, with a categorical omega of .92.

##### Work-Family Conflict

The Work-Family Conflict Scale (WAFCS; Haslam et al., 2015) was used to assess maternal work-to-family (WFC; 5 items) and family-to-work conflict (FWC; 5 items). Mothers indicated how much they agreed with 10 statements on a seven-point response format. For each subscale, the mean across five items was calculated, with higher scores indicating higher levels of work–family conflict. Reliabilities were high. Categorical omega was .87 for the WFC, and .89 for the FWC.

#### Analyses Plan

To assess the dimensionality of the questionnaire, Confirmatory Factor Analysis (CFA) and Exploratory Structural Equation Modeling (ESEM) with target rotation (Marsh et al., 2014) were performed in MPlus. For both, the ULSMV estimator was used (Forero et al., 2009). Model fit was evaluated with the root mean square error of approximation (RMSEA; value close to or below .06 indicates an adequate model fit), standardized root mean square residual (SRMR; value close to or below .08 indicates an adequate model fit), comparative fit index, and Tucker–Lewis index (CFI and TLI; value close to or above .95 indicates an adequate model fit) (Hu & Bentler, 1999). The possibility of a higher-order factor structure was examined as well.

The psychometric properties examined were reliability and construct validity (convergent and discriminant). Reliability and convergent validity analyses were performed in R 4.2.1; and discriminant validity analyses in MPlus. Categorical omegas (Green & Yang, 2009) were computed to assess the reliability (*misty v0.4.6*; Yanagida, 2022).

To determine whether the REWINd was related to the other measures of maternal well-being (convergent validity; Campbell & Fiske, 1959), bivariate correlations between maternal postpartum work resumption stress (overall score and subdomains) and maternal anxiety, postpartum depression, perceived stress, work-to-family conflict, and family-to-work conflict were examined. As individuals’ well-being is an important protective factor during life transitions (Schlossberg, 1981), mothers with lower levels of anxiety, depression, perceived stress, work-to-family conflict, and family-to-work conflict were expected to report lower levels of postpartum work resumption stress in general, and lower levels of *Work-life imbalance*, *Child-related concerns*, and *Lack of enrichment*, specifically.

To ensure that the REWINd measures constructs that are distinct from the other aspects of maternal well-being (discriminant validity; Rönkkö & Cho, 2022), a series of CFAs with two factors, one including items from the REWINd and the other including items from one of the other measures of well-being (i.e., anxiety, postpartum depression, perceived stress, work-to-family conflict, and family-to-work conflict) were conducted. Latent factors were scaled by fixing their variances to one. The absolute values of the upper/lower limit of the 95% confidence interval of the estimated factor correlations were inspected and compared against the cut-offs proposed by Rönkkö and Cho (2022).

### Results and Discussion

#### Questionnaire Dimensionality

The model with 30 items across three factors (i.e., *Work-life imbalance*, *Child-related concerns*, *Lack of enrichment*) was examined, to verify the factor structure previously found. There was no missing data. The model derived from the CFA, χ^2^(402, *N* = 291) = 949.40, *p* < .001, RMSEA = .07, CFI = .92, TLI = .92, SRMR = .06, showed somewhat adequate fit to the data. The model derived from the ESEM, χ^2^(348, *N* = 291) = 655.71, *p* < .001, RMSEA = .06, CFI = .96, TLI = .95, SRMR = .03, however showed good model fit. In both models, correlations between factors were positive and moderate (see Table S8 in Supplementary Material). As the ESEM model showed better model fit (Δχ^2^ = −293.69; Δ*df* = −54; ΔRMSEA = −.01; ΔCFI = .04; ΔTLI = .03; ΔSRMR = −.03), lower factor correlations (i.e., better discrimination between factors), and trivial cross-loadings (< .30) (van Zyl & Ten Klooster, 2022), this model was considered the best.

Next, by using an online H-ESEM code generator (de Beer & van Zyl, 2019), the higher-order structure was added to the ESEM model, with the three first-order factors loading on one second-order factor. Due to the just-identification of the model (i.e., no degrees of freedom for the second-order factor), we were unable to assess the fit of the model above the fit of the model with only first-order factors, and thus could not determine which model fits the data better. However, as all three first-order factors saliently and strongly loaded on the second-order factor, the higher order ESEM model was considered the final model in our analyses.

**Figure 1.**
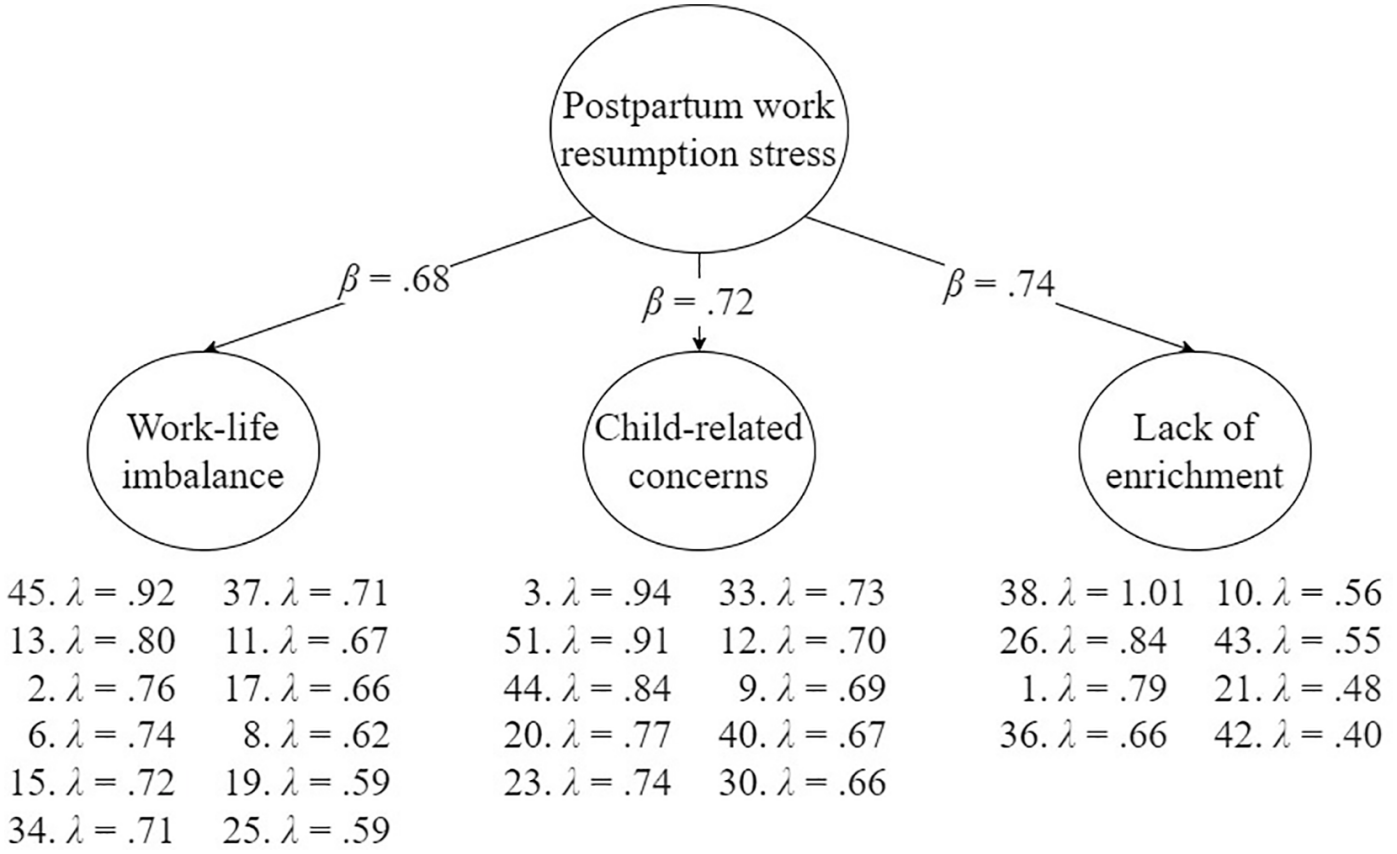
Standardized Factor Loadings (λ) of the Final (Higher-Order ESEM) Model.

Standardized factor loadings of the final model are shown in Figure 1. The results support the previously established dimensionality of our questionnaire. The three first-order factors (i.e., *Work-life imbalance*, *Child-related concerns*, *Lack of enrichment*) were well defined, with item loadings matching our expectations. All items saliently (>.30) loaded on one of the predefined factors, with factor loadings between .40 and 1.01. Cross-loadings were all below .30 and thus were considered trivial. The three first-order factors all saliently (>.30) and more or less equally strongly loaded on the higher-order factor, with loadings of .68, .72, and .74, respectively, providing evidence for a higher-order factor, capturing *the overall level of maternal postpartum work resumption stress*.

#### Reliability

All three first-order factors showed good reliability, with categorical omega of .93 for *Work–life imbalance*, .94 for *Child-related concerns*, and .88 for *Lack of enrichment*.

#### Convergent Validity

Bivariate correlations between the maternal postpartum work resumption stress variables and other aspects of maternal well-being are shown in Table 3. All correlations were significant and positive, ranging from .37 to .78. Consistent with our expectations, postpartum work resumption stress was related to other aspects of maternal well-being. Mothers with lower levels of anxiety, postpartum depression, perceived stress, work-to-family conflict, and family-to-work conflict showed lower levels of postpartum work resumption stress in general, as well as lower levels of *Work-life imbalance*, *Child-related concerns*, and *Lack of enrichment*—providing evidence for convergent validity of our questionnaire. In addition, partial correlations between the maternal postpartum work resumption stress variables and other aspects of maternal well-being, while controlling for maternal age, child age, maternal working hours, and length of leave separately, were performed (see Tables S9–S12 in Supplementary Material). Similar correlations were observed.

**Table 3:** Means, Standard Deviations, Minimum and Maximum Values, and Bivariate Correlations Across Maternal Well-being Variables.

Variable	Overall score	Work–life imbalance	Child-related concerns	Lack of enrichment	Anxiety	Postpartum depression	Perceived stress	Work-to-family conflict	Family-to-work conflict
Overall score	-								
Work–life imbalance	.834***	-							
Child-related concerns	.842***	.475***	-						
Lack of enrichment	.781***	.530***	.543***	-					
Anxiety^a^	.712***	.716***	.480***	.552***	-				
Postpartum depression	.633***	.647***	.446***	.445***	.750***	-			
Perceived stress^a^	.705***	.762***	.462***	.485***	.738***	.749***	-		
Work-to-family conflict	.782***	.663***	.656***	.596***	.555***	.505***	.563***	-	
Family-to-work conflict^a^	.530***	.506***	.413***	.365***	.445***	.478***	.547***	.470***	-
*M*	3.43	3.99^b^	3.25^b^	2.84^b^	2.02	.81	2.25	3.71	3.77
*SD*	.78	.90	1.10	.83	.53	.47	.46	1.17	1.29
Min	1.33	1.33	1.00	1.00	1.00	0.00	1.14	1.00	1.00
Max	5.53	6.00	5.90	5.50	3.65	2.40	3.71	7.00	7.00

*Note.* Overall score = Overall score of postpartum work resumption stress.

a Missingness occurred on the item level at the following variables and was highly limited: Anxiety, 1 item missing (*n* = 8), 2 items missing (*n* = 1), Perceived stress, 1 item missing (*n* = 1), 5 items missing (*n* = 1), and Family-to-work conflict, 1 item missing (*n* = 2). Person-mode imputation was used to calculate the item missing scores. ^b^Results from the repeated measures ANOVA (*rstatix v0.7.0*; Kassambara, 2021) showed significant differences between the levels of the three subdomains of postpartum work resumption stress, *F*(1.9, 547.8) = 220.5, *p* < .001, with post hoc analyses with Bonferroni adjustment showing significant differences between average levels of Child-related concerns and Lack of enrichment, *t*(290) = 7.3, *p* < .001, Child-related concerns and Work-life imbalance, *t*(290) = -12.2, *p* < .001, and Lack of enrichment and Work-life imbalance, *t*(290) = −23.4, *p* < .001.

*** *p* < .001.

#### Discriminant Validity

The absolute values of the upper/lower limit of the 95% confidence interval of the estimated factor correlations derived from CFAs among the maternal postpartum work resumption stress variables and other aspects of maternal well-being are shown in Table 4. In accordance with the cut-offs proposed by Rönkkö and Cho (2022), the absolute values of the upper/lower limit of the 95% confidence interval of the estimated factor correlations below .80 indicate no evidence of discriminant validity problem, the values below .90 a marginal problem, the values below 1.00 a moderate problem, and the values equal or above 1.00 a severe problem. Rönkkö and Cho (2022) further argue that while a marginal problem (value somewhere between .80 and .90) should be acknowledged, “the interpretation of scales as representations of a distinct construct is probably safe” (p. 31).

**Table 4:** The Absolute Value of the Upper/Lower Limit of the 95% CI of the Estimated Factor Correlations Derived from CFA.

Variable	Overall score	Work-life imbalance	Child-related concerns	Lack of enrichment
Anxiety^a^	.826^b^	.837^b^	.619^c^	.693^c^
Postpartum depression	.769^c^	.803^b^	.589^c^	.587^c^
Perceived stress^a^	.826^b^	.880^b^	.603^c^	.630^c^
Work-to-family conflict	.898^b^	.796^c^	.808^b^	.744^c^
Family-to-work conflict^a^	.667^c^	.640^c^	.566^c^	.506^c^

*Note.* Overall score = Overall score of maternal postpartum work resumption stress.

a Missingness occurred on the item level at the following variables and was highly limited: Anxiety, 1 item missing (*n* = 8), 2 items missing (*n* = 1), Perceived stress, 1 item missing (*n* = 1), 5 items missing (*n* = 1), and Family-to-work conflict, 1 item missing (*n* = 2). Person-mode imputation was used to calculate the item missing scores. ^b^ Marginal discriminant validity problem. ^c^No evidence of a discriminant validity problem.

Of the 20 absolute values of the upper/lower limit of the 95% confidence interval of the estimated factor correlations, 13 were below .80 and thus provided no evidence of a discriminant validity problem. The other seven values were between .80 and .90, and as such indicated a marginal discriminant validity problem. While no evidence of discriminant validity problems was observed for *Lack of enrichment*, a marginal problem with work-to-family conflict was found for *Child-related concerns*. The conceptual similarity between the two constructs might be due to the fact that both include items for negative consequences that being a working parent may have on one’s child.

Marginal discriminant validity problems were shown for *Work-life imbalance* as well, with respect to anxiety, postpartum depression, and perceived stress. An explanation might be that many *Work-life imbalance* items capture thoughts and emotions conceptually similar to items in the measures of anxiety, depression, and perceived stress, such as being tense, anxious, unbalanced, not being able to cope and not being in control. The conceptual similarities of *Child-related concerns* with work-to-family conflict and of *Work-life imbalance* with anxiety, depression, and perceived stress, might be the reason for the marginal validity problems between *overall levels of postpartum work resumption stress* and anxiety, perceived stress and work-to-family conflict.

For most correlations, there was no or only marginal evidence of discriminant validity problems. Therefore, preliminary evidence for the discriminant validity of our newly developed measure was found. While further validation is needed, our total scale and subscales can be considered to measure constructs that are distinct from other aspects of maternal well-being (anxiety, postpartum depression, perceived stress, work-to-family conflict, and family-to-work conflict).

## General Discussion

The goal of the current study was to advance our understanding of the stress mothers experience in response to returning to work after maternity leave. In accordance with previous qualitative and descriptive literature, we defined the concept of maternal postpartum work resumption stress as *an aversive psychological reaction to returning to work postpartum, that disrupts the individual’s homeostasis*, and developed a corresponding questionnaire measure. Based on exploratory and confirmatory analyses, a questionnaire with 30 items was established. Three subdomains of postpartum work resumption stress were differentiated: *Work-life imbalance*, *Child-related concerns*, and *Lack of enrichment*. Evidence for a higher-order factor, capturing the *overall level of maternal postpartum work resumption stress*, was found as well. As such, maternal levels of *Work-family imbalance*, *Child-related concerns*, and *Lack of enrichment* all importantly, and to a similar extent, contributed to the overall levels of stress mothers experience in response to returning to work after maternity leave. The questionnaire was shown to be a reliable and valid measure of maternal postpartum work resumption stress. We found support for convergent and discriminant validity, with the constructs related, yet distinct, from other aspects of maternal well-being, including anxiety, postpartum depression, perceived stress, work-to-family conflict, and family-to-work conflict.

### Theoretical Implications

With the development of our new measure, this study provides two important theoretical contributions. First, we gained further knowledge on how mothers in The Netherlands deal with the transition back to work after maternity leave. On average, Dutch mothers reported moderate levels of postpartum work resumption stress, with significant differences between the three subdomains (see Table 3). Compared with *Lack of enrichment* and *Child-related concerns*, mothers reported higher levels of *Work-life imbalance*. Furthermore, mothers reported significantly higher levels of *Child-related concerns* than *Lack of enrichment*. As such, while thoughts and emotions about lack of time, role-overload, and emotional and physical exhaustion when returning to work postpartum might be especially present among the Dutch mothers returning to work, ample positive thoughts and emotions about being back at work were experienced as well. This is in line with previous studies on postpartum return to work experience, showing mothers experiencing positive thoughts and emotions besides the negative (e.g., Alstveit et al., 2011; Costantini et al., 2022; Gallegos, 2007; Parcsi & Curtin, 2013; Spiteri & Borg Xuereb, 2012). In addition, this finding underscores the importance of seeing the transition back to work as enriching as well as challenging. Yet, further research with more demographically and clinically diverse samples is needed to generalize our findings and establish clinically significant cut-offs.

Second, the relation of postpartum work resumption stress with other well-being aspects was identified. Specifically, mothers reporting lower levels of postpartum work resumption stress in general, as well as lower levels of *Work–life imbalance*, *Child-related concerns*, and *Lack of enrichment* specifically, also reported lower levels of anxiety, postpartum depression, perceived stress, work-to-family conflict, and family-to-work conflict. Our data were cross-sectional, which means that causal direction could not be established; the findings indicate that maternal well-being might be an important protective factor or a distal outcome of stress when returning to work after maternity leave. Longitudinal and experimental intervention studies (e.g., by supporting mothers who return back to work) could shed further light on the causal relation between postpartum work resumption stress and maternal well-being. Such research is also important to identify other potential protective and risk factors for how mothers deal with the transition back to work after maternity leave (e.g., giving breastfeeding or not, having partner/social support or not).

### Strengths, Limitations, and Future Research

Our study is the first to define the construct of maternal postpartum work resumption stress and develop and validate a measure for it. While further research is needed to establish the robustness of our findings across different methodological aspects, we followed the most recent guidelines for best practices for developing and validating a new scale (e.g., Boateng et al., 2018; Carpenter, 2018; Goretzko et al., 2021; Marsh et al., 2014; Rönkkö & Cho, 2022; Watkins, 2018). We had the sample sizes needed for adequate power in the analyses (see Mundfrom et al., 2005; Wolf et al., 2013). Nevertheless, this study also had some limitations. First, the data were collected during the Covid-19 pandemic, limiting the generalizability of our findings to non-Covid-19 times. While only mothers were invited to participate who were physically separated from their children during work, the vast majority of our third sample (70.6%, *n* = 289) reported working from home for at least some number of their working hours. No information about the number of working hours from home was recorded for the second sample; however, one can assume that similar ratios of working from home occurred. As the experience of going back to work during the pandemic might differ from the experience in non-Covid-19 times, further validation of our questionnaire in non-pandemic times is warranted.

Second, the three samples of this study included mothers with a child who was not a twin, born pre-term or had developmental or health difficulties. Furthermore, despite the efforts made to include a more representative sample (i.e., Facebook ads targeting mothers in urban and rural areas across the Netherlands, with a specific focus on lower-educated mothers), participants in our study were mostly highly educated, living with a partner, and of Dutch nationality. Further studies with more clinically and demographically diverse samples are needed to determine the measurement invariance of our measure and ensure that our questionnaire is appropriate for other mothers as well (e.g., lower educated mothers, mothers in the Netherlands with non-Dutch nationality, single mothers, mothers with multiple pregnancies, and mothers of pre-term children or children with developmental difficulties). In addition, the focus of our study was on mothers from the Netherlands. Adjustment and validation of the questionnaire to other caregivers (e.g., fathers), adoptive parents, parents from non-traditional families, and parents from cultures with different leave policies, is needed for the generalization of our findings and broadening the questionnaire’s usability.

Third, as the majority of the reverse worded items loaded on the third factor the existence of the *Lack of enrichment* domain may be a reflection of the presence of the reverse wording as well. Specifically, in accordance with Zhang and colleagues (2016), the use of reverse items in Likert-type scales has potential drawbacks, as reverse-worded items can contaminate the factor structure of the scale, resulting in a more complex factor structure than necessary. Future research is needed to examine this possibility, for example, by using the Expanded format as an alternative to the Likert-type response format, which has been shown to reduce the impact of method effects on the structure of the scale (Zhang & Savalei, 2016). Nevertheless, we believe there is substantial literature and theory to support the existence of the *Lack of enrichment* domain. First, the qualitative and descriptive literature supports the idea that positive emotions and thoughts are present among mothers returning to work and that the non-existence of such emotions might result in stress. Second, in accordance with the Revised stress and coping model, positive emotions are a normative aspect of the stress process, responsible for restoring coping resources and sustaining coping (Folkman, 2008). As such, a lack of positive emotions in response to returning to work after maternity leave may contribute to the extent and persistence of the stress experience.

With the development of a measure of maternal postpartum work resumption stress, we hope to facilitate further research on postpartum return to work experience. The responses of individuals to transitions often change over time (Schlossberg, 2011). Therefore, studying changes in maternal postpartum work resumption stress throughout the transition back to work is warranted. Furthermore, further research is needed to establish the predictive validity of our questionnaire. For example, as the stress of individuals may trigger stress and strain in others in their social environment (Westman, 2001), the effect of maternal postpartum work resumption stress on other family members (e.g., infant, partner) should be studied. Moreover, employees’ well-being has been shown to be associated with one’s job performance (Gandy et al., 2014; Wright & Cropanzano, 2000). Thus, future studies should address the impact of maternal stress when returning to work postpartum on work-related outcomes. In addition, many aspects of maternal postpartum mental health, such as postpartum depression and anxiety, have been shown to be risk factors for various maternal and child outcomes (e.g., Field, 2018; Slomian et al., 2019). Seeing whether our newly developed measure has added value in predicting maternal and infant future health, over and above the already existing measures (i.e., incremental validity) is of importance.

As mentioned in the introduction, leave policies vary considerably across countries in terms of length of leave and benefits (OECD Family Database, 2022). Translating our questionnaire into other languages will make it possible to examine differences in maternal postpartum work resumption stress across various countries, and determine the impact of policies on maternal well-being. Finally, it has been suggested to study work-family enrichment as a potential buffer against negative consequences of work-family conflict (Peeters et al., 2013). For instance, experiencing high levels of enrichment when returning to work might counterbalance the negative impact of child-related concerns and work-life imbalance on work and family outcomes. Future research in needed to examine this idea.

### Societal Implications

In addition to the scientific relevance, our new questionnaire can contribute to societal advances. Prior research has shown that, upon the return to work after maternity leave, mental health problems might be the reason for mothers to take sick leave (van Beukering, 2002), report decreased productivity while at work (Uegaki et al., 2011), or voluntarily leave the organization altogether (Carlson et al., 2011). As productivity loss due to sick leave or decreased productivity while at work has been found to represent a considerable societal cost (Uegaki et al., 2011), and high levels of employee turnover can have negative consequences for the job leavers, the remaining workforce, as well as the overall performance of the organization (Mohammed et al., 2016), paying close attention to working mothers well-being as they make the transition back to work after maternity leave is of high societal and economical relevance. While further research is needed, our questionnaire shows promise to be a useful screening instrument to identify those mothers in need of support when returning to work after maternity leave. Specifically, assessing the three domains of postpartum work resumption stress (*Work-life imbalance*, *Child-related concerns*, *Lack of enrichment*), can help to identify the specific needs of mothers and give more personalized advice. Moreover, the REturn to Work INventory (REWINd) can be used to further study the nature, course, and consequences of maternal postpartum work resumption stress for mothers, as well as their families, and by doing so, provide support for advancing family policies for working mothers.

## Supplemental Material

sj-docx-1-asm-10.1177_10731911241246607 – Supplemental material for Maternal Postpartum Work Resumption Stress Supplemental material, sj-docx-1-asm-10.1177_10731911241246607 for Maternal Postpartum Work Resumption Stress by Ana Okorn, Madelon L. M. van Hooff, Antonius H. N. Cillessen and Roseriet Beijers in Assessment

## References

[bibr1-10731911241246607] Alstveit M. Severinsson E. Karlsen B. (2011). Readjusting one’s life in the tension inherent in work and motherhood. Journal of Advanced Nursing, 67(10), 2151–2160. 10.1111/j.1365-2648.2011.05660.x 21545634

[bibr2-10731911241246607] Beijers R. Bode L. Nout S. (2022). Werk hervatten na zwangerschapsverlof valt zwaar [Returning to work after maternity leave is difficult]. Vroeg, 39(1), 7–9.

[bibr3-10731911241246607] Boateng G. O. Neilands T. B. Frongillo E. A. Melgar-Quiñonez H. R. Young S. L. (2018). Best practices for developing and validating scales for health, social, and behavioral research: A primer. Frontiers in Public Health, 6, Article 149. 10.3389/fpubh.2018.00149 PMC6004510 29942800

[bibr4-10731911241246607] Brand H. Barreiro-Lucas J. (2014). Return-to-work experiences of female employees following maternity leave. South African Journal of Labour Relations, 38(1), 69–92.

[bibr5-10731911241246607] Brown T. A. (2015). Confirmatory factor analysis for applied research (2nd ed.). Guilford Press.

[bibr6-10731911241246607] Campbell D. T. Fiske D. W. (1959). Convergent and discriminant validation by the multitrait-multimethod matrix. Psychological Bulletin, 56, 81–105. 10.1037/h0046016 13634291

[bibr7-10731911241246607] Carlson D. S. Grzywacz J. G. Ferguson M. Hunter E. M. Clinch C. R. Arcury T. A. (2011). Health and turnover of working mothers after childbirth via the work-family interface: An analysis across time. Journal of Applied Psychology, 96(5), 1045–1054. 10.1037/a0023964 21604833 PMC3835182

[bibr8-10731911241246607] Carpenter S. (2018). Ten steps in scale development and reporting: A guide for researchers. Communication Methods and Measures, 12(1), 25–44. 10.1080/19312458.2017.1396583

[bibr9-10731911241246607] Cohen S. Kamarck T. Mermelstein R. (1983). A global measure of perceived stress. Journal of Health and Social Behavior, 24(4), 385–396. 10.2307/2136404 6668417

[bibr10-10731911241246607] Costantini A. Warasin R. Sartori R. Mantovan F. (2022). Return to work after prolonged maternity leave. An interpretative description. Women’s Studies International Forum, 90, 102562. 10.1016/j.wsif.2022.102562

[bibr11-10731911241246607] Costello A. B. Osborne J. W. (2005). Best practices in exploratory factor analysis: Four recommendations for getting the most from your analysis. Practical Assessment, Research and Evaluation, 10, 1–9. 10.7275/jyj1-4868

[bibr12-10731911241246607] Cox J. L. Holden J. M. Sagovsky R. (1987). Detection of postnatal depression: Development of the 10-item Edinburgh Postnatal Depression Scale. British Journal of Psychiatry, 150, 782–786. 10.1192/bjp.150.6.782 3651732

[bibr13-10731911241246607] Crosswell A. D. Lockwood K. G. (2020). Best practices for stress measurement: How to measure psychological stress in health research. Health Psychology Open, 7(2), 1–12. 10.1177/2055102920933072 PMC7359652 32704379

[bibr14-10731911241246607] de Beer L. T. van Zyl L. E . (2019). ESEM code generator for Mplus. https://www.surveyhost.co.za/esem/

[bibr15-10731911241246607] Ferketich S. (1991). Focus on psychometrics: Aspects of item analysis. Research in Nursing & Health, 14(2), 165–168. 10.1002/nur.4770140211 2047538

[bibr16-10731911241246607] Field T. (2018). Postnatal anxiety prevalence, predictors and effects on development: A narrative review. Infant Behavior & Development, 51, 24–32. 10.1016/j.infbeh.2018.02.005 29544195

[bibr17-10731911241246607] Folkman S. (2008). The case for positive emotions in the stress process. Anxiety, Stress, and Coping, 21(1), 3–14. 10.1080/10615800701740457 18027121

[bibr18-10731911241246607] Forero C. G. Maydeu-Olivares A. Gallardo-Pujol D. (2009). Factor analysis with ordinal indicators: A Monte Carlo study comparing DWLS and ULS estimation. Structural Equation Modeling, 16(4), 625–641. 10.1080/10705510903203573

[bibr19-10731911241246607] Gallegos D. (2007). Managing work and motherhood: Implications for perinatal mental health. State Perinatal Reference Group Department of Health, Centre for Social and Community Research, Western Australian Centre for Research for Women.

[bibr20-10731911241246607] Gandy W. M. Coberley C. Pope J. E. Wells A. Rula E. Y. (2014). Comparing the contributions of well-being and disease status to employee productivity. Journal of Occupational and Environmental Medicine, 56(3), 252–257. 10.1097/JOM.0000000000000109 24603200

[bibr21-10731911241246607] Garrido L. E. Abad F. J. Ponsoda V. (2013). A new look at Horn’s parallel analysis with ordinal variables. Psychological Methods, 18(4), 454–474. 10.1037/a0030005 23046000

[bibr22-10731911241246607] Geurts S. A. E. Taris T. W. Kompier M. A. J. Dikkers J. S. E. van Hooff M. L. M. Kinnunen U. M. (2005). Work-home interaction from a work psychological perspective: Development and validation of a new questionnaire, the SWING. Work & Stress, 19(4), 319–339. 10.1080/02678370500410208

[bibr23-10731911241246607] Goretzko D. Pham T. T. H. Bühner M. (2021). Exploratory factor analysis: Current use, methodological developments and recommendations for good practice. Current Psychology: A Journal for Diverse Perspectives on Diverse Psychological Issues, 40(7), 3510–3521. 10.1007/s12144-019-00300-2

[bibr24-10731911241246607] Green S. B. Yang Y. (2009). Reliability of summed item scores using structural equation modeling: An alternative to coefficient alpha. Psychometrika, 74, 155–167. 10.1007/s11336-008-9099-3

[bibr25-10731911241246607] Groer M. W. Davis M. W. Hemphill J. (2002). Postpartum stress: Current concepts and the possible protective role of breastfeeding. Journal of Obstetric, Gynecologic, and Neonatal Nursing, 31(4), 411–417. 10.1111/j.1552-6909.2002.tb00063.x 12146930

[bibr26-10731911241246607] Hair J. F. Jr. Black W. C. Babin B. J. Anderson R. E. (2009). Multivariate data analysis (7th ed.). Prentice Hall.

[bibr27-10731911241246607] Haslam D. Filus A. Morawska A. Sanders M. R. Fletcher R. (2015). The Work-Family Conflict Scale (WAFCS): Development and initial validation of a self-report measure of work-family conflict for use with parents. Child Psychiatry and Human Development, 46(3), 346–357. 10.1007/s10578-014-0476-0 24919779

[bibr28-10731911241246607] Haynes S. N. Richard D. C. S. Kubany E. S. (1995). Content validity in psychological assessment: A functional approach to concepts and methods. Psychological Assessment, 7(3), 238–247. 10.1037/1040-3590.7.3.238

[bibr29-10731911241246607] Hock E. McBride S. Gnezda M. T. (1989). Maternal separation anxiety: Mother-infant separation from the maternal perspective. Child Development, 60(4), 793–802. 10.2307/1131019

[bibr30-10731911241246607] Holden R. B. (2010). Face validity. In Weiner I. B. Craighead W. E. (Eds.), The Corsini encyclopedia of psychology (4th ed.). Wiley. 10.1002/9780470479216.corpsy0341

[bibr31-10731911241246607] Holm O. (1999). Analyses of longing: Origins, levels, and dimensions. The Journal of Psychology, 133(6), 621–630. 10.1080/00223989909599768

[bibr32-10731911241246607] Horowitz J. A. Damato E. G. (1999). Mother’s perceptions of postpartum stress and satisfaction. Journal of Obstetric, Gynecologic, and Neonatal Nursing, 28(6), 595–605. 10.1111/j.1552-6909.1999.tb02168.x 10584913

[bibr33-10731911241246607] Hu L. Bentler P. M. (1999). Cutoff criteria for fit indexes in covariance structure analysis: Conventional criteria versus new alternatives. Structural Equation Modeling, 6(1), 1–55.

[bibr34-10731911241246607] Jevitt C. M. Groer M. W. Crist N. F. Gonzalez L. Wagner V. D. (2012). Postpartum stressors: A content analysis. Issues in Mental Health Nursing, 33(5), 309–318. 10.3109/01612840.2011.653658 22545638

[bibr35-10731911241246607] Kaitz M. (2007). Maternal concerns during early parenthood. Child: Care, Health and Development, 33(6), 720–727. 10.1111/j.1365-2214.2007.00729.x 17944781

[bibr36-10731911241246607] Kassambara A. (2021). rstatix: Pipe-friendly framework for basic statistical tests (R Package Version 0.7.0). https://CRAN.R-project.org/package=rstatix

[bibr37-10731911241246607] Lazarus R. S. Folkman S. (1984). Stress, appraisal and coping. Springer.

[bibr38-10731911241246607] Lubbe D. (2019). Parallel analysis with categorical variables: Impact of category probability proportions on dimensionality assessment accuracy. Psychological Methods, 24(3), 339–351. 10.1037/met0000171 29745684

[bibr39-10731911241246607] Marsh H. W. Morin A. J. S. Parker P. D. Kaur G. (2014). Exploratory structural equation modeling: An integration of the best features of exploratory and confirmatory factor analysis. Annual Review of Clinical Psychology, 10(1), 85–110. 10.1146/annurev-clinpsy-032813-15370038 24313568

[bibr40-10731911241246607] Mikal J. P. Rice R. E. Abeyta A. DeVilbiss J. (2013). Transition, stress and computer-mediated social support. Computers in Human Behavior, 29(5), A40–A53. 10.1016/j.chb.2012.12.012

[bibr41-10731911241246607] Millward L. J. (2006). The transition to motherhood in an organizational context: An interpretative phenomenological analysis. Journal of Occupational and Organizational Psychology, 79, 315–333. 10.1348/096317906X110322

[bibr42-10731911241246607] Mohammed A.-M. Lai Y. Daskalaki M. Saridakis G. (2016). Employee turnover as a cost factor of organizations. In Saridakis G. Cooper C. L. (Eds.), Research handbook on employee turnover (pp. 109–126). Edward Elgar Publishing.

[bibr43-10731911241246607] Morris L. (2008). The experiences of women returning to work after maternity leave in the UK: Summary of survey results. http://www.nct.org.uk/sites/default/files/ReturningToWork-Survey.pdf

[bibr44-10731911241246607] Mundfrom D. J. Shaw D. G. Ke T. L. (2005). Minimum sample size recommendations for conducting factor analyses. International Journal of Testing, 5(2), 159–168. 10.1207/s15327574ijt0502_4

[bibr45-10731911241246607] Nichols M. R. Roux G. M. (2004). Maternal perspectives on postpartum return to the workplace. Journal of Obstetric, Gynecologic, and Neonatal Nursing, 33(4), 463–471. 10.1177/0884217504266909 15346672

[bibr46-10731911241246607] OECD Family Database. (2022, December). Parental leave systems. https://www.oecd.org/els/soc/PF2_1_Parental_leave_systems.pdf

[bibr47-10731911241246607] Parcsi L. Curtin M. (2013). Experiences of occupational therapists returning to work after maternity leave. Australian Occupational Therapy Journal, 60(4), 252–259. 10.1111/1440-1630.12051 23888975

[bibr48-10731911241246607] Peeters M. C. W. ten Brummelhuis L. L. van Steenbergen E. F. (2013). Consequences of combining work and family roles: A closer look at cross-domain versus within-domain relations. In Grzywacz J. G. Demerouti E. (Eds.), New frontiers in work and family research (pp. 93–109). Psychology Press.

[bibr49-10731911241246607] Polit D. F. Beck C. T. Owen S. V. (2007). Is the CVI an acceptable indicator of content validity? Appraisal and recommendations. Research in Nursing & Health, 30(4), 459–467. 10.1002/nur.20199 17654487

[bibr50-10731911241246607] Presaghi F. Desimoni M. (2020). random.polychor.pa: A Parallel Analysis with polychoric correlation matrices (R Package Version 1.1.4-4). https://CRAN.R-project.org/package=random.polychor.pa

[bibr51-10731911241246607] Razurel C. Bruchon-Schweitzer M. Dupanloup A. Irion O. Epiney M. (2011). Stressful events, social support and coping strategies of primiparous women during the postpartum period: A qualitative study. Midwifery, 27(2), 237–242. 10.1016/j.midw.2009.06.005 19783333

[bibr52-10731911241246607] Rönkkö M. Cho E. (2022). An updated guideline for assessing discriminant validity. Organizational Research Methods, 25(1), 6–14. 10.1177/1094428120968614

[bibr53-10731911241246607] Rosseel Y. (2012). lavaan: An R package for Structural Equation Modeling. Journal of Statistical Software, 48(2), 1–36. 10.18637/jss.v048.i02

[bibr54-10731911241246607] Ruscio J. (2018). RGenData: Generates multivariate nonnormal data and determines how many factors to retain (R Package Version 1.0). https://CRAN.R-project.org/package=RGenData

[bibr55-10731911241246607] Ruscio J. Roche B. (2012). Determining the number of factors to retain in an exploratory factor analysis using comparison data of known factorial structure. Psychological Assessment, 24(2), 282–292. 10.1037/a0025697 21966933

[bibr56-10731911241246607] Schlossberg N. K. (1981). A model for analyzing human adaptation to transition. The Counseling Psychologist, 9(2), 2–18. 10.1177/001100008100900202

[bibr57-10731911241246607] Schlossberg N. K. (2011). The challenge of change: The transition model and its applications. Journal of Employment Counseling, 48(4), 159–162. 10.1002/j.2161-1920.2011.tb01102.x

[bibr58-10731911241246607] Slomian J. Honvo G. Emonts P. Reginster J. Y. Bruyère O. (2019). Consequences of maternal postpartum depression: A systematic review of maternal and infant outcomes. Women’s Health, 15, 1–55. 10.1177/1745506519844044 PMC6492376 31035856

[bibr59-10731911241246607] Spielberger C. D. (1983). State-Trait Anxiety Inventory for Adults (STAI-AD) [Database record]. APA PsycTests. 10.1037/t06496-000

[bibr60-10731911241246607] Spiteri G. Borg Xuereb R. (2012). Going back to work after childbirth: Women’s lived experiences. Journal of Reproductive and Infant Psychology, 30(2), 201–216. 10.1080/02646838.2012.693153

[bibr61-10731911241246607] Steiner M. D. Grieder S. G. (2020). EFAtools: An R package with fast and flexible implementations of exploratory factor analysis tools. Journal of Open Source Software, 5(53), 2521. 10.21105/joss.02521

[bibr62-10731911241246607] Uegaki K. Stomp-van den Berg S. G. de Bruijne M. C. van Poppel M. N. M. Heymans M. W. van Mechelen W. van Tulder M. W. (2011). Cost-utility analysis of a one-time supervisor telephone contact at 6-weeks post-partum to prevent extended sick leave following maternity leave in The Netherlands: Results of an economic evaluation alongside a randomized controlled trial. BMC Public Health, 11, 57. 10.1186/1471-2458-11-57 21272325 PMC3040144

[bibr63-10731911241246607] van Beukering M. D. M . (2002). Werken tijdens zwangerschap en periode post-partum: Onderzoek naar ziekteverzuim [Working during pregnancy and the post-partum period: Research into absenteeism due to illness]. TBV, 10, 2–8.

[bibr64-10731911241246607] van Zyl L. E. Ten Klooster P. M . (2022). Exploratory Structural Equation Modeling: Practical guidelines and tutorial with a convenient online tool for Mplus. Frontiers in Psychiatry, 12, Article 795672. 10.3389/fpsyt.2021.795672 PMC8779472 35069293

[bibr65-10731911241246607] Watkins M. W. (2018). Exploratory factor analysis: A guide to best practice. Journal of Black Psychology, 44(3), 219–246. 10.1177/0095798418771807

[bibr66-10731911241246607] Westman M. (2001). Stress and strain crossover. Human Relations, 54, 557–591. 10.1177/0018726701546002

[bibr67-10731911241246607] Wolf E. J. Harrington K. M. Clark S. L. Miller M. W. (2013). Sample size requirements for structural equation models: An evaluation of power, bias, and solution propriety. Educational and Psychological Measurement, 73(6), 913–934. 10.1177/0013164413495237 PMC4334479 25705052

[bibr68-10731911241246607] Wright T. A. Cropanzano R. (2000). Psychological well-being and job satisfaction as predictors of job performance. Journal of Occupational Health Psychology, 5(1), 84–94. 10.1037/1076-8998.5.1.84 10658888

[bibr69-10731911241246607] Yanagida T. (2022). misty: Miscellaneous functions “T. Yanagida” (R Package Version 0.4.6). https://CRAN.R-project.org/package=misty

[bibr70-10731911241246607] Zhang X. Noor R. Savalei V. (2016). Examining the effect of reverse worded items on the factor structure of the Need for Cognition scale. PLOS ONE, 11(6), Article e0157795. 10.1371/journal.pone.0157795 PMC4909292 27305001

[bibr71-10731911241246607] Zhang X. Savalei V. (2016). Improving the factor structure of psychological scales: The expanded format as an alternative to the Likert scale format. Educational and Psychological Measurement, 76(3), 357–386. 10.1177/0013164415596421 27182074 PMC4849088

