# Supplementary material for: Maternal Postpartum Work Resumption Stress: Questionnaire Development and Validation
Source: Assessment. 2024 Apr 27;32(3):417–33. doi: 10.1177/10731911241246607 (PMC11915766; doi:10.1177/10731911241246607)
Supplement: sj-docx-1-asm-10.1177_10731911241246607 – Supplemental material for Maternal Postpartum Work Resumption Stress [file sj-docx-1-asm-10.1177_10731911241246607.docx]

**Table S1**

*EFA Results of the Three-Factor Solution*

| Item | *λ_1_* | *λ_2_* | *λ_3_* | *h^2^* |
| --- | --- | --- | --- | --- |
| *Now that I am back at work, …* | | | | |
| 1. I like having contact with people from work again. |  |  | **.68** | .49 |
| 2. I worry about myself. | **.67** |  |  | .54 |
| 3. I feel like I am letting my baby down. |  | **.85** |  | .77 |
| 4. I feel like I have to prove myself at work. | **.55** |  |  | .37 |
| 5. I feel I have control over my life. | **.53** |  | .35 | .54 |
| 6. I feel exhausted. | **.69** |  |  | .53 |
| 7. I feel like I take better care of my baby than anyone else. |  | **.44** |  | .21 |
| 8. I feel like I do not get the chance for my hobbies or to do other fun things. | **.70** |  |  | .49 |
| 9. I am afraid that the bond with my baby will worsen. |  | **.72** |  | .49 |
| 10. I feel ready to be back at work. |  | .32 | **.65** | .78 |
| 11. I feel like I have the energy to do everything I want. | **.56** |  |  | .49 |
| 12. I feel like I am falling short as a parent. |  | **.59** |  | .58 |
| 13. it is hard to find time for myself. | **.63** |  |  | .42 |
| 14. I can focus well on my work. | .47 |  | **.56** | .62 |
| 15. I feel stressed. | **.67** |  |  | .55 |
| 16. I worry about what people at work think of me now that I have a baby. | **.56** |  |  | .34 |
| 17. I struggle to meet the various responsibilities I have in my life. | **.76** |  |  | .55 |
| 18. I feel that my return to work has a positive impact on my baby. |  | .42 | **.48** | .41 |
| 19. I worry about my social life. | **.61** |  |  | .34 |
| 20. I am concerned that I will miss out on important moments in my baby’s life. |  | **.72** |  | .51 |
| 21. I feel like I have structure in my life. |  |  | **.42** | .39 |
| 22. I experience many positive emotions. | .30 |  | **.57** | .58 |
| 23. I am afraid that being back at work is not the best for my baby. |  | **.78** |  | .72 |
| 24. I worry that I am not involved enough in my work. | **.44** |  |  | .39 |
| 25. I feel overwhelmed by my role as a working parent. | **.62** |  |  | .57 |
| 26. my work gives me the opportunity to recharge myself. |  |  | **.61** | .39 |
| 27. I feel like I'm on my own. | **.45** |  |  | .41 |
| 28. I think my baby is happiest when he/she is cared for by me. |  | **.64** |  | .35 |
| 29. thoughts of work keep me from focusing on my family. | **.43** |  |  | .24 |
| 30. I am afraid that my baby will get sick, hurt, or upset while I am at work. |  | **.68** |  | .49 |
| 31. I feel that I function well at work. | .47 |  | **.58** | .70 |
| 32. I feel that my leave was long enough. |  | **.45** | .37 | .55 |
| 33. it feels too early to leave my baby in someone else’s care. |  | **.70** |  | .60 |
| 34. I feel like everything is chaotic. | **.71** |  |  | .56 |
| 35. I feel like I am a good parent. |  | **.42** |  | .28 |
| 36. I lack the motivation to do my job. |  |  | **.62** | .60 |
| 37. I struggle managing my time well. | **.77** |  |  | .53 |
| 38. I enjoy being back at work. |  |  | **.79** | .78 |
| 39. I'm having a hard time getting used to being back at work. |  |  | **.39** | .52 |
| 40. I feel like I have no control over my baby’s care and upbringing. |  | **.58** |  | .48 |
| 41. I worry that I am a burden to the people from my work. | **.46** |  |  | .31 |
| 42. my life feels complete. |  |  | **.51** | .43 |
| 43. I feel proud as a working parent. |  |  | **.65** | .51 |
| 44. I feel like I am not with my baby often enough. |  | **.70** |  | .57 |
| 45. I feel like I do not have enough time to take care of myself. | **.75** |  |  | .58 |
| 46. I have no trouble adapting to my work. |  |  | **.34** | .24 |
| 47. I worry that people around me think I'm a bad parent. |  | **.42** |  | .27 |
| 48. I have trouble remembering things. | **.53** |  |  | .28 |
| 49. I don't worry much about my baby when I'm at work. |  | **.40** |  | .20 |
| 50. I feel that my return to work has a negative impact on relationships with my loved ones. | **.51** |  |  | .47 |
| 51. I feel guilty for leaving my baby with others. |  | **.91** |  | .80 |
| 52. I feel that I am effective at work. | **.54** |  | .49 | .62 |
| *ω* | .95 | .95 | .94 |  |

*Note.* *λ_1_* = geomin factor loading on the first factor; *λ_2_* = geomin factor loading on the second factor; *λ_3_* = geomin factor loading on the third factor; *h^2^* = item communality; *ω* = categorical omega (the highest loading was used to decide on the factor the item is part of). Bold = item highest loading. Non-salient loadings (<. 30) are omitted for clarity.

**Table S2**

*EFA Results of the Six-Factor Solution*

| Item | *λ_1_* | *λ_2_* | *λ_3_* | *λ_4_* | *λ_5_* | *λ_6_* | *h^2^* |
| --- | --- | --- | --- | --- | --- | --- | --- |
| *Now that I am back at work, …* | | | | | | | |
| 1. I like having contact with people from work again. | -.31 |  | **.80** |  |  |  | .53 |
| 2. I worry about myself. | **.51** |  |  |  |  |  | .55 |
| 3. I feel like I am letting my baby down. |  | **.70** |  |  |  |  | .78 |
| 4. I feel like I have to prove myself at work. |  |  |  |  | .34 | **.39** | .49 |
| 5. I feel I have control over my life. | .40 |  | **.43** |  |  |  | .56 |
| 6. I feel exhausted. | **.62** |  |  |  |  |  | .57 |
| 7. I feel like I take better care of my baby than anyone else. |  |  |  | **.69** |  |  | .56 |
| 8. I feel like I do not get the chance for my hobbies or to do other fun things. | **.74** |  |  |  |  |  | .58 |
| 9. I am afraid that the bond with my baby will worsen. |  | **.80** |  |  |  |  | .65 |
| 10. I feel ready to be back at work. |  |  | **.67** |  |  |  | .78 |
| 11. I feel like I have the energy to do everything I want. | **.54** |  |  |  |  |  | .55 |
| 12. I feel like I am falling short as a parent. |  | **.57** |  |  |  |  | .61 |
| 13. it is hard to find time for myself. | **.80** |  |  |  |  |  | .61 |
| 14. I can focus well on my work. |  |  | .44 |  | **.67** |  | .80 |
| 15. I feel stressed. | **.56** |  |  |  |  |  | .58 |
| 16. I worry about what people at work think of me now that I have a baby. |  |  |  |  |  | **.66** | .62 |
| 17. I struggle to meet the various responsibilities I have in my life. | **.63** |  |  |  |  |  | .57 |
| 18. I feel that my return to work has a positive impact on my baby. |  |  | **.55** | .40 |  |  | .51 |
| 19. I worry about my social life. | **.57** |  |  |  |  |  | .35 |
| 20. I am concerned that I will miss out on important moments in my baby’s life. |  | **.84** |  |  |  |  | .61 |
| 21. I feel like I have structure in my life. |  |  | **.44** |  |  |  | .44 |
| 22. I experience many positive emotions. |  |  | **.70** |  |  |  | .63 |
| 23. I am afraid that being back at work is not the best for my baby. |  | **.58** |  |  |  |  | .71 |
| 24. I worry that I am not involved enough in my work. |  |  |  |  | **.51** |  | .51 |
| 25. I feel overwhelmed by my role as a working parent. | **.53** |  |  |  |  |  | .59 |
| 26. my work gives me the opportunity to recharge myself. |  |  | **.69** |  |  |  | .42 |
| 27. I feel like I'm on my own. | **.44** |  |  |  |  |  | .44 |
| 28. I think my baby is happiest when he/she is cared for by me. |  |  |  | **.73** |  |  | .66 |
| 29. thoughts of work keep me from focusing on my family. |  |  |  |  |  | **.43** | .37 |
| 30. I am afraid that my baby will get sick, hurt, or upset while I am at work. |  | **.50** |  |  |  |  | .55 |
| 31. I feel that I function well at work. |  |  | .54 |  | **.59** |  | .81 |
| 32. I feel that my leave was long enough. |  | .34 | **.39** |  |  |  | .59 |
| 33. it feels too early to leave my baby in someone else’s care. |  | **.50** |  | .32 |  |  | .64 |
| 34. I feel like everything is chaotic. | **.56** |  |  |  |  |  | .56 |
| 35. I feel like I am a good parent. |  | **.41** |  |  |  |  | .37 |
| 36. I lack the motivation to do my job. |  |  | **.61** |  | .42 |  | .66 |
| 37. I struggle managing my time well. | **.62** |  |  |  |  |  | .55 |
| 38. I enjoy being back at work. |  |  | **.90** |  |  |  | .81 |
| 39. I'm having a hard time getting used to being back at work. |  |  | **.39** |  |  |  | .54 |
| 40. I feel like I have no control over my baby’s care and upbringing. |  | **.53** |  |  |  |  | .54 |
| 41. I worry that I am a burden to the people from my work. |  |  |  |  |  | **.54** | .51 |
| 42. my life feels complete. |  |  | **.63** |  |  |  | .52 |
| 43. I feel proud as a working parent. |  |  | **.77** |  |  |  | .58 |
| 44. I feel like I am not with my baby often enough. |  | **.62** |  |  |  |  | .58 |
| 45. I feel like I do not have enough time to take care of myself. | **.72** |  |  |  |  |  | .63 |
| 46. I have no trouble adapting to my work. |  |  | **.35** |  |  |  | .24 |
| 47. I worry that people around me think I'm a bad parent. |  | **.43** |  |  |  | .34 | .36 |
| 48. I have trouble remembering things. | **.37** |  |  |  | .32 |  | .34 |
| 49. I don't worry much about my baby when I'm at work. |  | **.38** |  |  |  |  | .23 |
| 50. I feel that my return to work has a negative impact on relationships with my loved ones. | **.35** |  |  |  |  | .30 | .56 |
| 51. I feel guilty for leaving my baby with others. |  | **.80** |  |  |  |  | .82 |
| 52. I feel that I am effective at work. |  |  | .40 |  | **.66** |  | .78 |
| *ω* | .93 | .92 | .93 | -^a^ | .88 | .73 |  |

*Note.* *λ_1_* = geomin factor loading on the first factor; *λ_2_* = geomin factor loading on the second factor; *λ_3_* = geomin factor loading on the third factor; *λ_4_* = geomin factor loading on the fourth factor; *λ_5_* = geomin factor loading on the fifth factor; *λ_6_* = geomin factor loading on the sixth factor; *h^2^* = item communality; *ω* = categorical omega (the highest loading was used to decide on the factor the item is part of). Bold = item highest loading. Non-salient loadings (<. 30) are omitted for clarity. The solution was considered inadequate due to underdetermination and thus possible instability of the fourth and fifth factor. Specifically, although four items had a salient loading on the fourth factor, only two of them loaded uniquely on this factor. Similarly, from the seven items that loaded saliently on the fifth factor, only one loaded uniquely on this factor. Thus, only two items loaded uniquely on the fourth factor and only one on the fifth.

^a^At least three items are needed for computing omega.

**Table S3**

*EFA Results of the Five-Factor Solution*

| Item | *λ_1_* | *λ_2_* | *λ_3_* | *λ_4_* | *λ_5_* | *h^2^* |
| --- | --- | --- | --- | --- | --- | --- |
| *Now that I am back at work, …* |  |  |  |  |  |  |
| 1. I like having contact with people from work again. | **.74** |  |  |  |  | .52 |
| 2. I worry about myself. |  | **.54** |  |  |  | .55 |
| 3. I feel like I am letting my baby down. |  |  | **.82** |  |  | .77 |
| 4. I feel like I have to prove myself at work. |  |  |  | **.46** |  | .46 |
| 5. I feel I have control over my life. | .40 | **.45** |  |  |  | .57 |
| 6. I feel exhausted. |  | **.64** |  |  |  | .56 |
| 7. I feel like I take better care of my baby than anyone else. |  |  | **.64** |  | -.44 | .47 |
| 8. I feel like I do not get the chance for my hobbies or to do other fun things. |  | **.77** |  |  |  | .58 |
| 9. I am afraid that the bond with my baby will worsen. |  |  | **.59** |  | .44 | .63 |
| 10. I feel ready to be back at work. | **.71** |  |  |  |  | .78 |
| 11. I feel like I have the energy to do everything I want. | .32 | **.56** |  |  |  | .54 |
| 12. I feel like I am falling short as a parent. |  |  | **.49** |  |  | .60 |
| 13. it is hard to find time for myself. |  | **.83** |  |  |  | .61 |
| 14. I can focus well on my work. | **.66** |  |  | **.53** |  | .75 |
| 15. I feel stressed. |  | **.61** |  |  |  | .58 |
| 16. I worry about what people at work think of me now that I have a baby. |  |  |  | **.43** | .36 | .51 |
| 17. I struggle to meet the various responsibilities I have in my life. |  | **.66** |  |  |  | .56 |
| 18. I feel that my return to work has a positive impact on my baby. | **.47** |  | .44 |  |  | .48 |
| 19. I worry about my social life. |  | **.60** |  |  |  | .35 |
| 20. I am concerned that I will miss out on important moments in my baby’s life. |  |  | **.63** |  |  | .51 |
| 21. I feel like I have structure in my life. | **.46** |  |  |  |  | .43 |
| 22. I experience many positive emotions. | **.64** |  |  |  |  | .61 |
| 23. I am afraid that being back at work is not the best for my baby. |  |  | **.73** |  |  | .72 |
| 24. I worry that I am not involved enough in my work. | .37 |  |  | **.53** |  | .52 |
| 25. I feel overwhelmed by my role as a working parent. |  | **.55** |  |  |  | .59 |
| 26. my work gives me the opportunity to recharge myself. | **.63** |  |  |  |  | .40 |
| 27. I feel like I'm on my own. |  | **.48** |  |  |  | .43 |
| 28. I think my baby is happiest when he/she is cared for by me. |  |  | **.78** |  | -.42 | .56 |
| 29. thoughts of work keep me from focusing on my family. |  |  |  |  | **.34** | .30 |
| 30. I am afraid that my baby will get sick, hurt, or upset while I am at work. |  |  | **.71** |  |  | .55 |
| 31. I feel that I function well at work. | **.70** |  |  | .52 |  | .81 |
| 32. I feel that my leave was long enough. | .39 |  | **.42** |  |  | .59 |
| 33. it feels too early to leave my baby in someone else’s care. |  |  | **.70** |  |  | .65 |
| 34. I feel like everything is chaotic. |  | **.59** |  |  |  | .56 |
| 35. I feel like I am a good parent. |  |  |  |  | **.39** | .38 |
| 36. I lack the motivation to do my job. | **.72** |  |  | .35 |  | .66 |
| 37. I struggle managing my time well. |  | **.64** |  |  |  | .54 |
| 38. I enjoy being back at work. | **.84** |  |  |  |  | .78 |
| 39. I'm having a hard time getting used to being back at work. | **.45** |  |  |  |  | .54 |
| 40. I feel like I have no control over my baby’s care and upbringing. |  |  | **.56** |  |  | .54 |
| 41. I worry that I am a burden to the people from my work. |  |  |  | **.43** |  | .46 |
| 42. my life feels complete. | **.56** |  |  |  |  | .53 |
| 43. I feel proud as a working parent. | **.67** |  |  |  |  | .54 |
| 44. I feel like I am not with my baby often enough. |  |  | **.65** |  |  | .57 |
| 45. I feel like I do not have enough time to take care of myself. |  | **.75** |  |  |  | .63 |
| 46. I have no trouble adapting to my work. | **.39** |  |  |  |  | .25 |
| 47. I worry that people around me think I'm a bad parent. |  |  | .32 |  | **.40** | .36 |
| 48. I have trouble remembering things. |  | **.37** |  |  |  | .29 |
| 49. I don't worry much about my baby when I'm at work. |  |  | **.42** |  |  | .21 |
| 50. I feel that my return to work has a negative impact on relationships with my loved ones. |  | **.40** |  |  | .39 | .56 |
| 51. I feel guilty for leaving my baby with others. |  |  | **.86** |  |  | .80 |
| 52. I feel that I am effective at work. | **.61** |  |  | .56 |  | .74 |
| *ω* | .96 | .94 | .95 | .75 | .48 |  |

*Note.* *λ_1_* = geomin factor loading on the first factor; *λ_2_* = geomin factor loading on the second factor; *λ_3_* = geomin factor loading on the third factor; *λ_4_* = geomin factor loading on the fourth factor; *λ_5_* = geomin factor loading on the fifth factor; *h^2^* = item communality; *ω* = categorical omega (the highest loading was used to decide on the factor the item is part of). Bold = item highest loading. Non-salient loadings (<. 30) are omitted for clarity. The solution was considered inadequate due to underdetermination and thus possible instability of the fourth and fifth factor. Specifically, although eight items had a salient loading on the fourth factor, only two of them loaded uniquely on this factor. Similarly, from the eight items that loaded saliently on the fifth factor, only two loaded uniquely on this factor. Thus, only two items loaded uniquely on the fourth and fifth factor. Furthermore, the reliability of the fifth factor was unacceptable (categorical omega = .48).

**Table S4**

*EFA Results of the Four-Factor Solution*

| Item | *λ_1_* | *λ_2_* | *λ_3_* | *λ_4_* | *h^2^* |
| --- | --- | --- | --- | --- | --- |
| *Now that I am back at work, …* |  |  |  |  |  |
| 1. I like having contact with people from work again. | **.77** |  | -.38 |  | .50 |
| 2. I worry about myself. |  |  | **.51** |  | .54 |
| 3. I feel like I am letting my baby down. |  | **.82** |  |  | .77 |
| 4. I feel like I have to prove myself at work. |  |  |  | **.41** | .43 |
| 5. I feel I have control over my life. | **.45** |  | .38 |  | .54 |
| 6. I feel exhausted. |  |  | **.67** |  | .56 |
| 7. I feel like I take better care of my baby than anyone else. |  | **.42** |  |  | .23 |
| 8. I feel like I do not get the chance for my hobbies or to do other fun things. |  |  | **.81** |  | .58 |
| 9. I am afraid that the bond with my baby will worsen. |  | **.74** |  | .33 | .58 |
| 10. I feel ready to be back at work. | **.75** |  |  |  | .78 |
| 11. I feel like I have the energy to do everything I want. | .35 |  | **.57** |  | .54 |
| 12. I feel like I am falling short as a parent. |  | **.57** |  |  | .58 |
| 13. it is hard to find time for myself. |  |  | **.87** |  | .62 |
| 14. I can focus well on my work. | **.72** |  |  |  | .65 |
| 15. I feel stressed. |  |  | **.56** |  | .54 |
| 16. I worry about what people at work think of me now that I have a baby. |  |  |  | **.59** | .52 |
| 17. I struggle to meet the various responsibilities I have in my life. |  |  | **.66** |  | .55 |
| 18. I feel that my return to work has a positive impact on my baby. | **.48** | .35 |  | -.31 | .47 |
| 19. I worry about my social life. |  |  | **.59** |  | .34 |
| 20. I am concerned that I will miss out on important moments in my baby’s life. |  | **.69** |  |  | .51 |
| 21. I feel like I have structure in my life. | **.50** |  |  |  | .39 |
| 22. I experience many positive emotions. | **.69** |  |  |  | .58 |
| 23. I am afraid that being back at work is not the best for my baby. |  | **.74** |  |  | .72 |
| 24. I worry that I am not involved enough in my work. | **.42** |  |  | .34 | .44 |
| 25. I feel overwhelmed by my role as a working parent. |  |  | **.58** |  | .58 |
| 26. my work gives me the opportunity to recharge myself. | **.67** |  |  |  | .39 |
| 27. I feel like I'm on my own. |  |  | **.45** |  | .42 |
| 28. I think my baby is happiest when he/she is cared for by me. |  | **.61** |  | -.30 | .43 |
| 29. thoughts of work keep me from focusing on my family. |  |  |  |  | .26 |
| 30. I am afraid that my baby will get sick, hurt, or upset while I am at work. |  | **.68** |  |  | .51 |
| 31. I feel that I function well at work. | **.77** |  |  | .32 | .76 |
| 32. I feel that my leave was long enough. | **.40** | .39 |  |  | .60 |
| 33. it feels too early to leave my baby in someone else’s care. |  | **.65** |  |  | .65 |
| 34. I feel like everything is chaotic. |  |  | **.60** |  | .56 |
| 35. I feel like I am a good parent. |  | **.42** |  |  | .29 |
| 36. I lack the motivation to do my job. | **.78** |  |  |  | .64 |
| 37. I struggle managing my time well. |  |  | **.67** |  | .54 |
| 38. I enjoy being back at work. | **.89** |  |  |  | .78 |
| 39. I'm having a hard time getting used to being back at work. | **.50** |  |  |  | .53 |
| 40. I feel like I have no control over my baby’s care and upbringing. |  | **.59** |  | .31 | .54 |
| 41. I worry that I am a burden to the people from my work. |  |  |  | **.54** | .46 |
| 42. my life feels complete. | **.58** |  |  |  | .44 |
| 43. I feel proud as a working parent. | **.71** |  |  |  | .53 |
| 44. I feel like I am not with my baby often enough. |  | **.68** |  |  | .57 |
| 45. I feel like I do not have enough time to take care of myself. |  |  | **.78** |  | .63 |
| 46. I have no trouble adapting to my work. | **.43** |  |  |  | .24 |
| 47. I worry that people around me think I'm a bad parent. |  | **.45** |  | .37 | .34 |
| 48. I have trouble remembering things. |  |  | **.41** |  | .28 |
| 49. I don't worry much about my baby when I'm at work. |  | **.38** |  |  | .20 |
| 50. I feel that my return to work has a negative impact on relationships with my loved ones. |  |  | **.31** |  | .48 |
| 51. I feel guilty for leaving my baby with others. |  | **.87** |  |  | .80 |
| 52. I feel that I am effective at work. | **.68** |  |  | .35 | .68 |
| *ω* | .97 | .93 | .93 | .73 |  |

*Note.* *λ_1_* = geomin factor loading on the first factor; *λ_2_* = geomin factor loading on the second factor; *λ_3_* = geomin factor loading on the third factor; *λ_4_* = geomin factor loading on the fourth factor; *h^2^* = item communality; *ω* = categorical omega (the highest loading was used to decide on the factor the item is part of). Bold = item highest loading. Non-salient loadings (<. 30) are omitted for clarity. The solution seemed adequate at first (e.g., at least three items loaded uniquely on each of the four factors). But further examination showed that the fourth factor was unstable. Specifically, after excluding item 29, that did not load saliently on any factors, of the nine items with a salient loading on the fourth factor, only two items loaded uniquely on this factor, deeming the factor weak and unstable.

**Table S5**

*EFA Results of the Final Solution with Oblimin Rotation*

| Item | *λ_1_* | *λ_2_* | *λ_3_* | *h^2^* |
| --- | --- | --- | --- | --- |
| **Factor 1: Work-Life Imbalance** |  |  |  |  |
| *Now that I am back at work, …* | | | | |
| 45. I feel like I do not have enough time to take care of myself. | .80 |  |  | .64 |
| 37. I struggle managing my time well. | .77 |  |  | .55 |
| 8. I feel like I do not get the chance for my hobbies or to do other fun things. | .76 |  |  | .55 |
| 17. I struggle to meet the various responsibilities I have in my life. | .76 |  |  | .55 |
| 13. it is hard to find time for myself. | .75 |  |  | .55 |
| 34. I feel like everything is chaotic. | .71 |  |  | .57 |
| 6. I feel exhausted. | .70 |  |  | .54 |
| 11. I feel like I have the energy to do everything I want. | .64 |  |  | .52 |
| 25. I feel overwhelmed by my role as a working parent. | .63 |  |  | .59 |
| 15. I feel stressed. | .63 |  |  | .51 |
| 2. I worry about myself. | .62 |  |  | .51 |
| 19. I worry about my social life. | .56 |  |  | .32 |
| **Factor 2: Child-Related Concerns** | | | | |
| *Now that I am back at work, …* | | | | |
| 51. I feel guilty for leaving my baby with others. |  | .91 |  | .81 |
| 3. I feel like I am letting my baby down. |  | .85 |  | .77 |
| 9. I am afraid that the bond with my baby will worsen. |  | .80 |  | .53 |
| 20. I am concerned that I will miss out on important moments in my baby’s life. |  | .77 |  | .56 |
| 44. I feel like I am not with my baby often enough. |  | .74 |  | .60 |
| 23. I am afraid that being back at work is not the best for my baby. |  | .73 |  | .70 |
| 30. I am afraid that my baby will get sick, hurt, or upset while I am at work. |  | .68 |  | .46 |
| 40. I feel like I have no control over my baby’s care and upbringing. |  | .65 |  | .49 |
| 33. it feels too early to leave my baby in someone else’s care. |  | .60 |  | .55 |
| 12. I feel like I am falling short as a parent. |  | .58 |  | .59 |
| **Factor 3: Lack of Enrichment** | | | | |
| *Now that I am back at work, …* | | | | |
| 38. I enjoy being back at work. |  |  | .90 | .85 |
| 1. I like having contact with people from work again. |  |  | .80 | .57 |
| 43. I feel proud as a working parent. |  |  | .73 | .56 |
| 10. I feel ready to be back at work. |  |  | .67 | .76 |
| 26. my work gives me the opportunity to recharge myself. |  |  | .65 | .40 |
| 36. I lack the motivation to do my job. |  |  | .63 | .55 |
| 42. my life feels complete. |  |  | .56 | .44 |
| 21. I feel like I have structure in my life. |  |  | .46 | .40 |

*Note.* *λ_1_* = oblimin factor loading on the first factor; *λ_2_* = oblimin factor loading on the second factor; *λ_3_* = oblimin factor loading on the third factor; *h^2^* = item communality. Non-salient loadings (<. 30) are omitted for clarity. The solution obtained is the same as the solution obtained with geomin rotation, supporting the stability and appropriateness of our final solution.

**Table S6**

*EFA Results of the Final Solution with Promax Rotation*

| Item | *λ_1_* | *λ_2_* | *λ_3_* | *h^2^* |
| --- | --- | --- | --- | --- |
| **Factor 1: Work-Life Imbalance** |  |  |  |  |
| *Now that I am back at work, …* | | | | |
| 45. I feel like I do not have enough time to take care of myself. |  | .82 |  | .64 |
| 37. I struggle managing my time well. |  | .79 |  | .55 |
| 8. I feel like I do not get the chance for my hobbies or to do other fun things. |  | .78 |  | .55 |
| 17. I struggle to meet the various responsibilities I have in my life. |  | .78 |  | .55 |
| 13. it is hard to find time for myself. |  | .77 |  | .55 |
| 34. I feel like everything is chaotic. |  | .72 |  | .57 |
| 6. I feel exhausted. |  | .71 |  | .54 |
| 25. I feel overwhelmed by my role as a working parent. |  | .64 |  | .59 |
| 11. I feel like I have the energy to do everything I want. |  | .64 |  | .52 |
| 15. I feel stressed. |  | .64 |  | .51 |
| 2. I worry about myself. |  | .63 |  | .51 |
| 19. I worry about my social life. |  | .59 |  | .32 |
| **Factor 2: Child-Related Concerns** | | | | |
| *Now that I am back at work, …* | | | | |
| 51. I feel guilty for leaving my baby with others. | .91 |  |  | .81 |
| 3. I feel like I am letting my baby down. | .84 |  |  | .77 |
| 9. I am afraid that the bond with my baby will worsen. | .80 |  |  | .53 |
| 20. I am concerned that I will miss out on important moments in my baby’s life. | .77 |  |  | .56 |
| 44. I feel like I am not with my baby often enough. | .74 |  |  | .60 |
| 23. I am afraid that being back at work is not the best for my baby. | .73 |  |  | .70 |
| 30. I am afraid that my baby will get sick, hurt, or upset while I am at work. | .68 |  |  | .46 |
| 40. I feel like I have no control over my baby’s care and upbringing. | .64 |  |  | .49 |
| 33. it feels too early to leave my baby in someone else’s care. | .60 |  |  | .55 |
| 12. I feel like I am falling short as a parent. | .57 |  |  | .59 |
| **Factor 3: Lack of Enrichment** | | | | |
| *Now that I am back at work, …* | | | | |
| 38. I enjoy being back at work. |  |  | .92 | .85 |
| 1. I like having contact with people from work again. |  |  | .82 | .57 |
| 43. I feel proud as a working parent. |  |  | .74 | .56 |
| 10. I feel ready to be back at work. |  |  | .68 | .76 |
| 26. my work gives me the opportunity to recharge myself. |  |  | .66 | .40 |
| 36. I lack the motivation to do my job. |  |  | .64 | .55 |
| 42. my life feels complete. |  |  | .57 | .44 |
| 21. I feel like I have structure in my life. |  |  | .46 | .40 |

*Note.* *λ_1_* = promax factor loading on the first factor; *λ_2_* = promax factor loading on the second factor; *λ_3_* = promax factor loading on the third factor; *h^2^* = item communality. Non-salient loadings (<. 30) are omitted for clarity. The solution obtained is the same as the solution obtained with geomin rotation, supporting the stability and appropriateness of our final solution.

**Table S7**

*EFA Results of the Final Solution with Quartimin Rotation*

| Item | *λ_1_* | *λ_2_* | *λ_3_* | *h^2^* |
| --- | --- | --- | --- | --- |
| **Factor 1: Work-Life Imbalance** |  |  |  |  |
| *Now that I am back at work, …* | | | | |
| 45. I feel like I do not have enough time to take care of myself. | .80 |  |  | .64 |
| 37. I struggle managing my time well. | .77 |  |  | .55 |
| 8. I feel like I do not get the chance for my hobbies or to do other fun things. | .76 |  |  | .55 |
| 17. I struggle to meet the various responsibilities I have in my life. | .76 |  |  | .55 |
| 13. it is hard to find time for myself. | .75 |  |  | .55 |
| 34. I feel like everything is chaotic. | .71 |  |  | .57 |
| 6. I feel exhausted. | .70 |  |  | .54 |
| 11. I feel like I have the energy to do everything I want. | .64 |  |  | .52 |
| 25. I feel overwhelmed by my role as a working parent. | .63 |  |  | .59 |
| 15. I feel stressed. | .63 |  |  | .51 |
| 2. I worry about myself. | .62 |  |  | .51 |
| 19. I worry about my social life. | .56 |  |  | .32 |
| **Factor 2: Child-Related Concerns** | | | | |
| *Now that I am back at work, …* | | | | |
| 51. I feel guilty for leaving my baby with others. |  | .91 |  | .81 |
| 3. I feel like I am letting my baby down. |  | .85 |  | .77 |
| 9. I am afraid that the bond with my baby will worsen. |  | .80 |  | .53 |
| 20. I am concerned that I will miss out on important moments in my baby’s life. |  | .77 |  | .56 |
| 44. I feel like I am not with my baby often enough. |  | .74 |  | .60 |
| 23. I am afraid that being back at work is not the best for my baby. |  | .73 |  | .70 |
| 30. I am afraid that my baby will get sick, hurt, or upset while I am at work. |  | .68 |  | .46 |
| 40. I feel like I have no control over my baby’s care and upbringing. |  | .65 |  | .49 |
| 33. it feels too early to leave my baby in someone else’s care. |  | .60 |  | .55 |
| 12. I feel like I am falling short as a parent. |  | .58 |  | .59 |
| **Factor 3: Lack of Enrichment** | | | | |
| *Now that I am back at work, …* | | | | |
| 38. I enjoy being back at work. |  |  | .90 | .85 |
| 1. I like having contact with people from work again. |  |  | .80 | .57 |
| 43. I feel proud as a working parent. |  |  | .73 | .56 |
| 10. I feel ready to be back at work. |  |  | .67 | .76 |
| 26. my work gives me the opportunity to recharge myself. |  |  | .65 | .40 |
| 36. I lack the motivation to do my job. |  |  | .63 | .55 |
| 42. my life feels complete. |  |  | .56 | .44 |
| 21. I feel like I have structure in my life. |  |  | .46 | .40 |

*Note.* *λ_1_* = quartimin factor loading on the first factor; *λ_2_* = quartimin factor loading on the second factor; *λ_3_* = quartimin factor loading on the third factor; *h^2^* = item communality. Non-salient loadings (<. 30) are omitted for clarity. The solution obtained is the same as the solution obtained with geomin rotation, supporting the stability and appropriateness of our final solution.

**Table S8**

*Factor Correlations in CFA and ESEM Model*

| Factor | CFA model | | ESEM model | |
| --- | --- | --- | --- | --- |
|  | Factor 1 | Factor 2 | Factor 1 | Factor 2 |
| Factor 1 | - |  | - |  |
| Factor 2 | .55 | - | .49 | - |
| Factor 3 | .62 | .62 | .50 | .53 |

**Table S9**

*Partial Correlations across Maternal Well-being Variables: Controlling for the Effect of Maternal Age*

|  | Overall score | Work-life imbalance | Child-related concerns | Lack of enrichment | Anxiety | Postpartum depression | Perceived stress | Work-to-family conflict | Family-to-work conflict |
| --- | --- | --- | --- | --- | --- | --- | --- | --- | --- |
| Work-life imbalance | .834*** | - |  |  |  |  |  |  |  |
| Child-related concerns | .842*** | .475*** | - |  |  |  |  |  |  |
| Lack of enrichment | .782*** | .531*** | .546*** | - |  |  |  |  |  |
| Anxiety^a^ | .712*** | .716*** | .480*** | .554*** | - |  |  |  |  |
| Postpartum depression | .633*** | .647*** | .446*** | .446*** | .750*** | - |  |  |  |
| Perceived stress^a^ | .706*** | .763*** | .461*** | .489*** | .738*** | .749*** | - |  |  |
| Work-to-family conflict | .784*** | .665*** | .660*** | .594*** | .557*** | .507*** | .568*** | - |  |
| Family-to-work conflict^a^ | .531*** | .507*** | .415*** | .362*** | .446*** | .479*** | .551*** | .468*** | - |

*Note.* Overall score = Overall score of postpartum work resumption stress.

^a^Missingness occurred on the item level at the following variables and was highly limited: Anxiety (1 item missing (*n* = 8), 2 items missing (*n* = 1)), Perceived stress (1 item missing (*n* = 1), 5 items missing (*n* = 1)), and Family-to-work conflict (1 item missing (*n* = 2)). Person-mode imputation was used to calculate the item missing scores. ****p* < .001.

**Table S10**

*Partial Correlations across Maternal Well-being Variables: Controlling for the Effect of Child Age*

|  | Overall score | Work-life imbalance | Child-related concerns | Lack of enrichment | Anxiety | Postpartum depression | Perceived stress | Work-to-family conflict | Family-to-work conflict |
| --- | --- | --- | --- | --- | --- | --- | --- | --- | --- |
| Work-life imbalance | .840*** | - |  |  |  |  |  |  |  |
| Child-related concerns | .847*** | .495*** | - |  |  |  |  |  |  |
| Lack of enrichment | .776*** | .522*** | .548*** | - |  |  |  |  |  |
| Anxiety^a^ | .708*** | .713*** | .487*** | .541*** | - |  |  |  |  |
| Postpartum depression | .637*** | .643*** | .468*** | .435*** | .749*** | - |  |  |  |
| Perceived stress^a^ | .713*** | .761*** | .483*** | .485*** | .741*** | .751*** | - |  |  |
| Work-to-family conflict | .779*** | .661*** | .665*** | .587*** | .548*** | .498*** | .562*** | - |  |
| Family-to-work conflict^a^ | .541*** | .500*** | .448*** | .363*** | .444*** | .476*** | .543*** | .472*** | - |

*Note.* Overall score = Overall score of postpartum work resumption stress. Due to missing values on child age, data is based on a subsample of 287 participants. ^a^Missingness occurred on the item level at the following variables and was highly limited: Anxiety (1 item missing (*n* = 8), 2 items missing (*n* = 1)), Perceived stress (1 item missing (*n* = 1), 5 items missing (*n* = 1)), and Family-to-work conflict (1 item missing (*n* = 2)). Person-mode imputation was used to calculate the item missing scores. ****p* < .001.

**Table S11**

*Partial Correlations across Maternal Well-being Variables: Controlling for the Effect of Maternal Work Hours*

|  | Overall score | Work-life imbalance | Child-related concerns | Lack of enrichment | Anxiety | Postpartum depression | Perceived stress | Work-to-family conflict | Family-to-work conflict |
| --- | --- | --- | --- | --- | --- | --- | --- | --- | --- |
| Work-life imbalance | .833*** | - |  |  |  |  |  |  |  |
| Child-related concerns | .840*** | .470*** | - |  |  |  |  |  |  |
| Lack of enrichment | .775*** | .526*** | .536*** | - |  |  |  |  |  |
| Anxiety^a^ | .708*** | .714*** | .474*** | .546*** | - |  |  |  |  |
| Postpartum depression | .630*** | .644*** | .441*** | .438*** | .748*** | - |  |  |  |
| Perceived stress^a^ | .701*** | .760*** | .455*** | .476*** | .735*** | .747*** | - |  |  |
| Work-to-family conflict | .795*** | .668*** | .661*** | .619*** | .560*** | .508*** | .569*** | - |  |
| Family-to-work conflict^a^ | .524*** | .502*** | .407*** | .353*** | .439*** | .473*** | .543*** | .474*** | - |

*Note.* Overall score = Overall score of postpartum work resumption stress.

^a^Missingness occurred on the item level at the following variables and was highly limited: Anxiety (1 item missing (*n* = 8), 2 items missing (*n* = 1)), Perceived stress (1 item missing (*n* = 1), 5 items missing (*n* = 1)), and Family-to-work conflict (1 item missing (*n* = 2)). Person-mode imputation was used to calculate the item missing scores. ****p* < .001.

**Table S12**

*Partial Correlations across Maternal Well-being Variables: Controlling for the Effect of Length of Leave*

|  | Overall score | Work-life imbalance | Child-related concerns | Lack of enrichment | Anxiety | Postpartum depression | Perceived stress | Work-to-family conflict | Family-to-work conflict |
| --- | --- | --- | --- | --- | --- | --- | --- | --- | --- |
| Work-life imbalance | .839*** | - |  |  |  |  |  |  |  |
| Child-related concerns | .839*** | .479*** | - |  |  |  |  |  |  |
| Lack of enrichment | .779*** | .538*** | .533*** | - |  |  |  |  |  |
| Anxiety^a^ | .708*** | .715*** | .474*** | .550*** | - |  |  |  |  |
| Postpartum depression | .630*** | .644*** | .443*** | .441*** | .747*** | - |  |  |  |
| Perceived stress^a^ | .711*** | .762*** | .469*** | .494*** | .740*** | .749*** | - |  |  |
| Work-to-family conflict | .782*** | .662*** | .659*** | .595*** | .551*** | .500*** | .563*** | - |  |
| Family-to-work conflict^a^ | .538*** | .509*** | .423*** | .373*** | .450*** | .481*** | .548*** | .472*** | - |

*Note.* Overall score = Overall score of postpartum work resumption stress. Due to missing values on length of leave variable, data is based on a subsample of 288 participants. ^a^Missingness occurred on the item level at the following variables and was highly limited: Anxiety (1 item missing (*n* = 8), 2 items missing (*n* = 1)), Perceived stress (1 item missing (*n* = 1), 5 items missing (*n* = 1)), and Family-to-work conflict (1 item missing (*n* = 2)). Person-mode imputation was used to calculate the item missing scores. ****p* < .001.

**Figure S1**

*Flow chart depicting the procedure taken to arrive to the final version of the questionnaire*

**
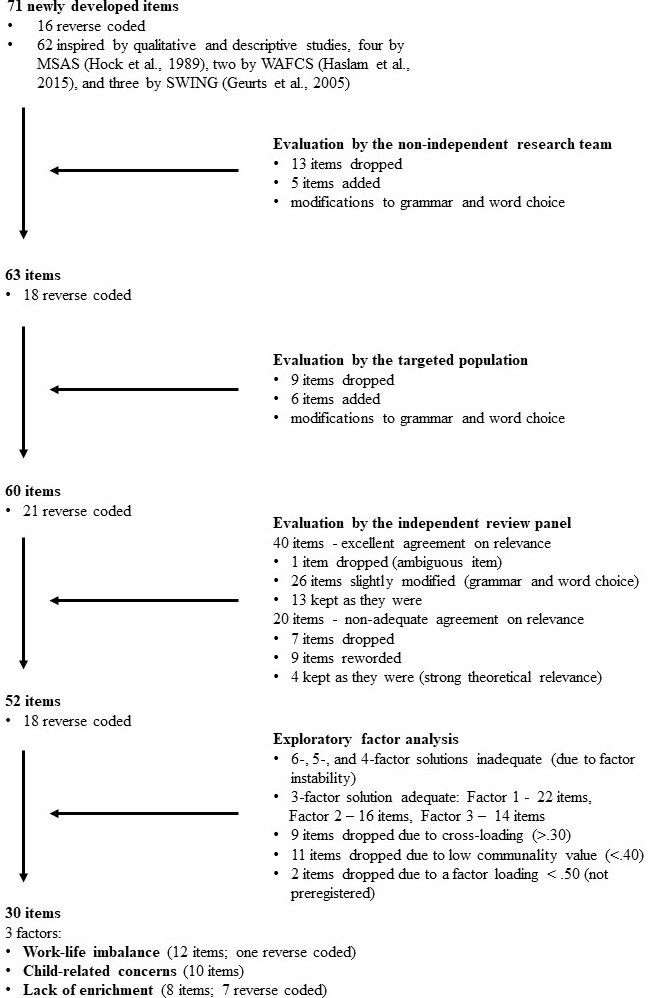
**

**Figure S2**

*Inter-item Polychoric Correlations Among 52 Items*

*
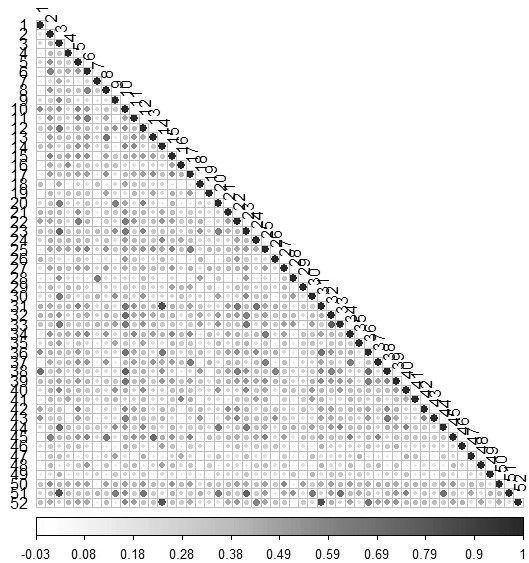
*
